# The SlDOF9‐SlSWEET17 Module: a Switch for Controlling Sugar Distribution Between Nematode Induced Galls and Roots in Tomato

**DOI:** 10.1002/advs.202501771

**Published:** 2025-05-11

**Authors:** Xiaoyun Wang, Zhimei Wang, Xinyue Tang, Jiamei Qin, Xiaoxuan Zhou, Lixia Gu, Huihui Bian, Lulu Sun, Huang Huang, Rui Yang, Jianli Wang, Shaohui Wang, Shuangchen Chen, Zhongren Yang, Wenchao Zhao

**Affiliations:** ^1^ College of Plant Science and Technology Beijing University of Agriculture No. 7 Beinong Road, Changping District Beijing 102206 China; ^2^ Beijing Key Laboratory for Agricultural Application and New Technique Beijing University of Agriculture Beijing 102206 China; ^3^ College of Horticulture and Plant Protection Henan University of Science and Technology Luoyang 471023 China; ^4^ College of Horticulture and Plant Protection Inner Mongolia Agricultural University Inner Mongolia 010010 China

**Keywords:** DOF transcription factor, meloidogyne incognita, *Solanum lycopersicum* L., sugar distribution, SWEET protein

## Abstract

In the complex interactions between plants and pathogens, the regulation of nutrient allocation plays a critical role in determining plant health and susceptibility to diseases. Root‐knot nematodes (RKNs, *Meloidogyne incognita*) extract sugar from plants during their interactions with the hosts. SWEET (Sugars Will Eventually be Exported Transporters) proteins are a class of non‐energy‐consuming sugar uniporters that regulate the allocation of sugars in plant. Here, it is find that SlSWEET17 (*Solanum lycopersicum* SWEET17), a member of the SWEET family in tomato, is localized to the plasma membrane, Golgi body and small vacuoles, and is highly expressed in galls. Further studies show that SlSWEET17 negatively regulates the sugar transport capacity of other SlSWEETs via protein interactions. Overexpression of *SlSWEET17* significantly decreases the soluble sugar content in galls and susceptibility to RKNs, while *SlSWEET17* knockout‐mutation (ko‐mutation) has the opposite effect. It is also identified SlDOF9 (*Solanum lycopersicum* DNA binding with one finger 9), an upstream negative regulator of SlSWEET17, using ChIP (chromatin immunoprecipitation) analysis, electrophoretic mobility shift assays and dual‐Luciferase assays. *SlDOF9‐*overexpressing plants show increased sugar content in galls and susceptibility to RKNs, and *sl* *dof9^cr^* ko‐mutants have the opposite phenotype. This results show how SlDOF9‐SlSWEET17 affects RKN infection through sugar partitioning from roots to galls.

## Graphical abstract

Root‐knot nematode (RKN) disease seriously affects the yield and quality of vegetable crops. SlDOF9‐SlSWEET17 model helps plants resist RKN infection during early stage by switching off the sugar transport capacity of other SlSWEET proteins that are hijacked by RKNs. This study provides new insights into the molecular mechanisms of sugar distribution and crop susceptibility to pathogen mediated by SWEETs.

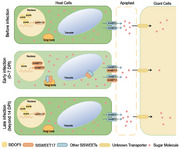

## 1 Introduction

As autotrophs, plants can synthesize nutrients, mainly sugars, through photosynthesis to maintain growth and development. Root pathogens like nematodes survive as heterotrophs by obtaining sugars from the hosts.^[^ ^1^ ^]^ During evolution, plants and pathogens often engage in a “tug‐of‐war”, with pathogens evolving adaptive strategies to acquire sugars from the hosts, while plants restrict pathogens from acquiring sugars and initiating immune responses. Therefore, regulating sugar distribution between plants and pathogens may be a key host defense strategy to limit pathogen proliferation.^[^ ^2^ ^]^

Root‐knot nematodes (RKNs, *Meloidogyne incognita*), which are a group of sedentary endoparasitic nematodes species, strongly affect the yield and quality of crops.^[^ ^3^ ^]^ As parasitic organisms, RKNs mainly obtain nutrients from their host plants. Under suitable environmental conditions, they pierce the plant root cortex to enter the vascular cylinder and select six to eight parenchymal cells to induce the formation of specialized, multinucleated, overexpanded, metabolically active giant cells (GCs). The GCs and the surrounding tissue, including the xylem and phloem cells, formed *de novo*, proliferate rapidly and eventually form a gall (also known as root knot), which is the main symptom of RKN disease.^[^ ^4^ ^]^ Mature GCs serve as feeding sites for RKNs and take up essential nutrients, such as sugars, from extracellular space (apoplast) of plant roots. Both the plants and pathogens maneuver for apoplastic sugar supplies into a competitive arms race for energy,^[^ ^5^ ^]^ but the mechanism of competition for apoplastic sugar between plants and RKNs is not clear.

Plants stringently control apoplastic sugar level by sugar transporters and glycoside hydrolases.^[^ ^2^ ^]^ SUT/SUC (Sucrose transporter/Sugar transporter)‐type transporters primarily import sucrose from the cell wall into the cells for long‐distance transport, and SWEETs (Sugars will eventually exported via transporters) mediate sucrose efflux from putative phloem parenchyma into the phloem apoplasm, as a key step proceeding phloem loading.^[^ ^6^ ^]^ SWEETs proteins are typically predicted to have seven transmembrane domains and can transport oligosaccharides bidirectionally across membranes along concentration gradients, without energy consumption and pH‐independent.^[^ ^7^ ^]^ Xuan et al. used the transient expression system of tobacco leaves to prove that SWEETs can form homooligomerization, which is essential for the function of their sugar transporters.^[^ ^8^ ^]^

Pathogens can alter the direction of sugar flow by hijacking the nutrient efflux mechanism of the host for their own nutrients.^[^ ^5^ ^]^ Studies have shown that members of the SWEET family are the targets of pathogens.^[^ ^6^ ^]^ Recent studies have shown that *Xanthomonas oryzae* pv. *oryzae* (*Xoo*) can secrete PthXo2 and PthXo2‐like proteins, which are type III TAL (Transcription Activator Like) effectors that can directly bind to the promoter of rice *OsSWEET11/13/14* genes. Thereby they activate transcription of sugar transporter‐encoding genes and promote sugar efflux into the apoplasm and parasites for nutritional gain.^[^ ^9^ ^]^ In *Arabidopsis thaliana*, AtSWEET11 and AtSWEET12 are upregulated by *Ustilaginoidea virus* and *Plasmodiophora brassicae*, respectively, and negatively regulate disease resistance.^[^ ^10^ ^]^ Recently, it was reported that the parasitism of RKNs was significantly impaired in *atsweet1* and *atsweet10* mutants compared to wild‐type plants in *Arabidopsis thaliana*, probably implying that SWEET proteins might be one of the sugar transport pathways hijacked by RKNs.^[^ ^11^ ^]^ However, the regulatory mechanisms of the SWEET family in sugar transport between plants and RKNs remain unclear.

To date, there have been few reports about upstream transcription factors regulating *SWEET* genes. In cotton, the transcription factor GhMYB212 directly binds to the promoter of *GhSWEET12* and regulates its transcription, thereby affecting cotton fiber elongation.^[^ ^12^ ^]^ The pear transcription factor PuWRKY31 binds to the promoter of *PuSWEET15* and affects sucrose accumulation in fruits.^[^ ^13^ ^]^ Additionally, the rice OsDOF11 transcription factor can bind to the *OsSWEET14* promoter, regulate sugar transport, and subsequently change rice susceptibility to *Xanthomonas oryzae* pathovar *oryzae* and *Rhizoctonia solani*.^[^ ^14^ ^]^ Recently, studies have demonstrated that the *Arabidopsis* transcription factor AtHB24 affects the sugar supply to the roots and root growth under salt stress by regulating *AtSWEET11/12*.^[^ ^15^ ^]^ In general, further studies on the upstream regulatory mechanism of *SWEET* genes during pathogenesis are needed to clearly understand the plant‐RKN interactions.

In this work, we observe that *SlSWEET17* (*Solanum lycopersicum SWEET17*) is upregulated in response to RKN parasitism but negatively regulates sugar accumulation in galls and tomato susceptibility to RKNs. In addition, SlDOF9, an upstream regulator, mediates expression of the *SlSWEET17* gene in galls and coregulates susceptibility to RKNs. Here, we show that the SlDOF9‐SlSWEET17 module serves as a sugar transport switch during nematode infection and plays a positive regulatory role in plant‐RKN interactions. This study improves our understanding of the molecular mechanisms by which the host restricts sugar distribution in galls.

## 2 Results

### 2.1 Tissue and Subcellular Localization of SlSWEET17

**Figure 1:**
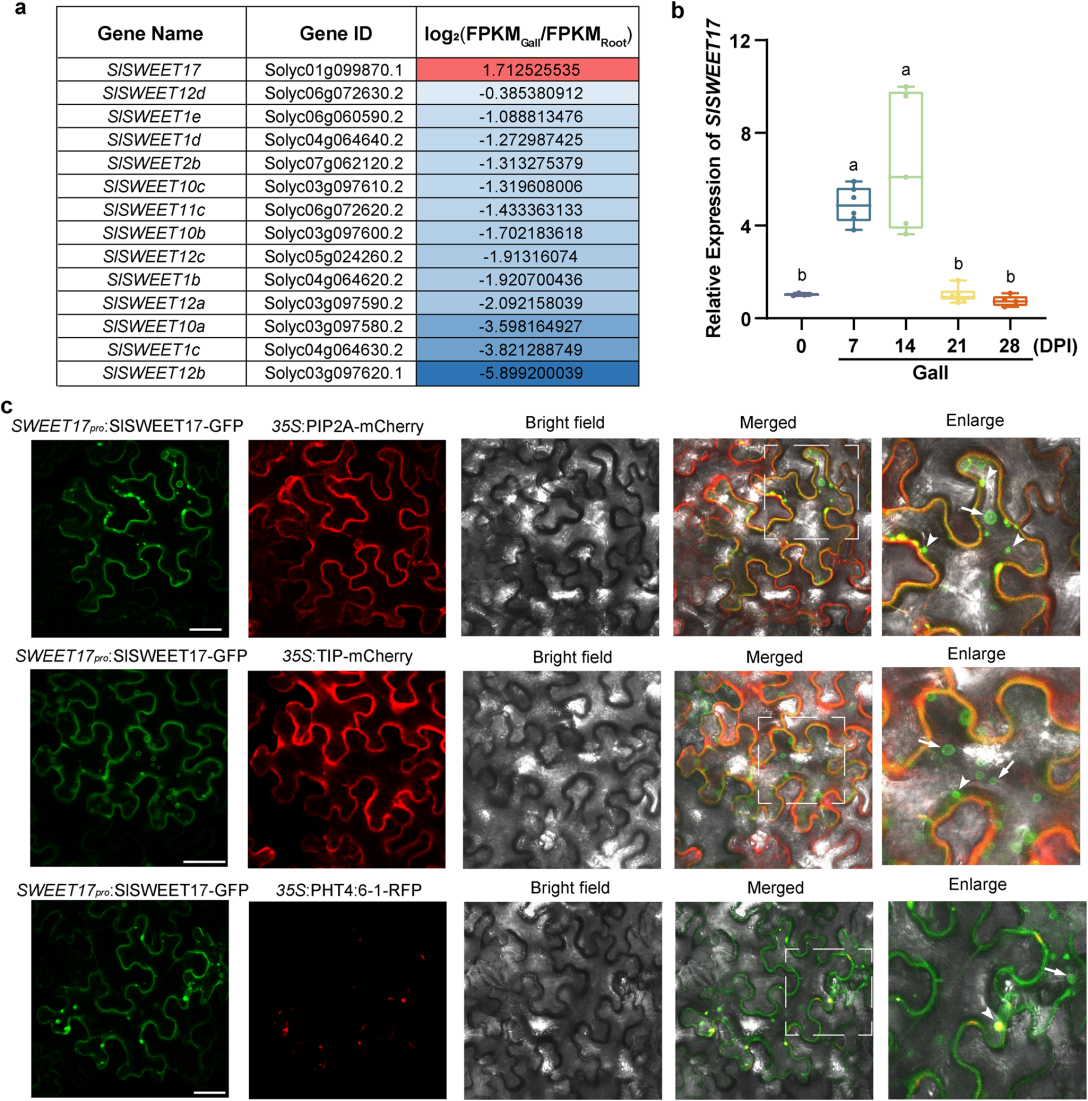
SlSWEET17 highly expressed in galls and localized to the plasma membrane, vacuoles and Golgi bodies. a) Changes in *SlSWEET* genes expression in galls versus roots. The expression of *SWEET* genes was tested by RNA‐Seq, and the changes were indicated by log_2_(FPKM_Gall_/FPKM_Root_). b) The relative expression of *SlSWEET17* of multiple time points (including 0, 7,14,21,28 DPI) during RKN infection. Box plots indicate the median (horizontal line), the interquartile range from 25% to 75%, and whiskers indicate the ±SE of at least four biological replicates. Different letters indicate significant differences as determined by ANOVA followed by Tukey's multiple range test. c) Subcellular localization of SlSWEET17 driven by *SlSWEET17* promoter in *N. benthamiana*, respectively. *35S*:PIP2A‐mCherry was used as plasma membrane marker. *35S*:TIP‐mCherry was used as tonoplast marker. *35S*:PHT4:6‐1‐RFP was used as Golgi bodies marker. White boxes denote the locations of the enlarged images, which are shown at the right. Arrows indicate vacuoles, and tailless arrows indicate Golgi bodies. Bar = 40 µm. (SWEET, sugars will eventually be exported transporters; RNA‐Seq, RNA sequencing; FPKM, fragments per kilobase of transcript per million fragments mapped; DPI, day post inoculation; pro, protomer; PIP2A, plasma membrane intrinsic protein 2A; TIP, to noplast intrinsic protein; PHT, phosphate transporter; GFP, green fluorescence protein; RFP, red fluorescence protein).

To identify SWEET genes that influence sugar partitioning into galls 14 days post RKN inoculation (DPI), the uninoculated and inoculated root systems were used for transcriptome analysis. A total of 7007 differentially expressed genes (DEGs) were identified (Table S1, Supporting Information). Considering the role of *SWEET* genes in pathogen‐plant interactions and sugar unloading processes,^[^ ^16^ ^]^ *SWEET* genes expressed in galls were screened for subsequent analysis. The expression of most *SlSWEET* genes was downregulated, while *SlSWEET17* was significantly upregulated (2.1‐fold) (**Figure** **1**a). Next, quantitative real‐time polymerase chain reaction (qRT‐PCR) was used to quantify *SlSWEET17* gene expression at 0,7, 14, 21, and 28 DPI in galls (Figure 1b). The results showed that the expression level of *SlSWEET17* significantly increased in the early stage of RKN infection. Next, SlSWEET17 localization was studied via transient overexpression of *SWEET17_pro_*:SlSWEET17‐GFP fusion protein in tobacco leaves. The fusion protein co‐localized with plasma membrane marker PIP2A‐mCherry, tonoplast marker TIP‐mCherry and Golgi body marker PHT4:6‐1‐RFP (Figure 1c). To further confirm the subcellular localization of SlSWEET17 in galls, tomato cotyledons were transformed to produce hairy roots for RKN inoculation using *Agrobacterium rhizogenes* carrying the *SWEET17_pro_*: SlSWEET17‐GFP vector.^[^ ^7b^ ^]^ GFP fluorescence was mainly localized in galls at 2 DPI and 4 DPI, and was barely detected in gall‐adjacent tissues. The enlarged area showed that SlSWEET17 was mainly localized in and around the plasma membrane in 4 DPI galls (Figure S1, Supporting Information).

### 2.2 SlSWEET17 Interacts with Multiple SlSWEET Proteins

**Figure 2:**
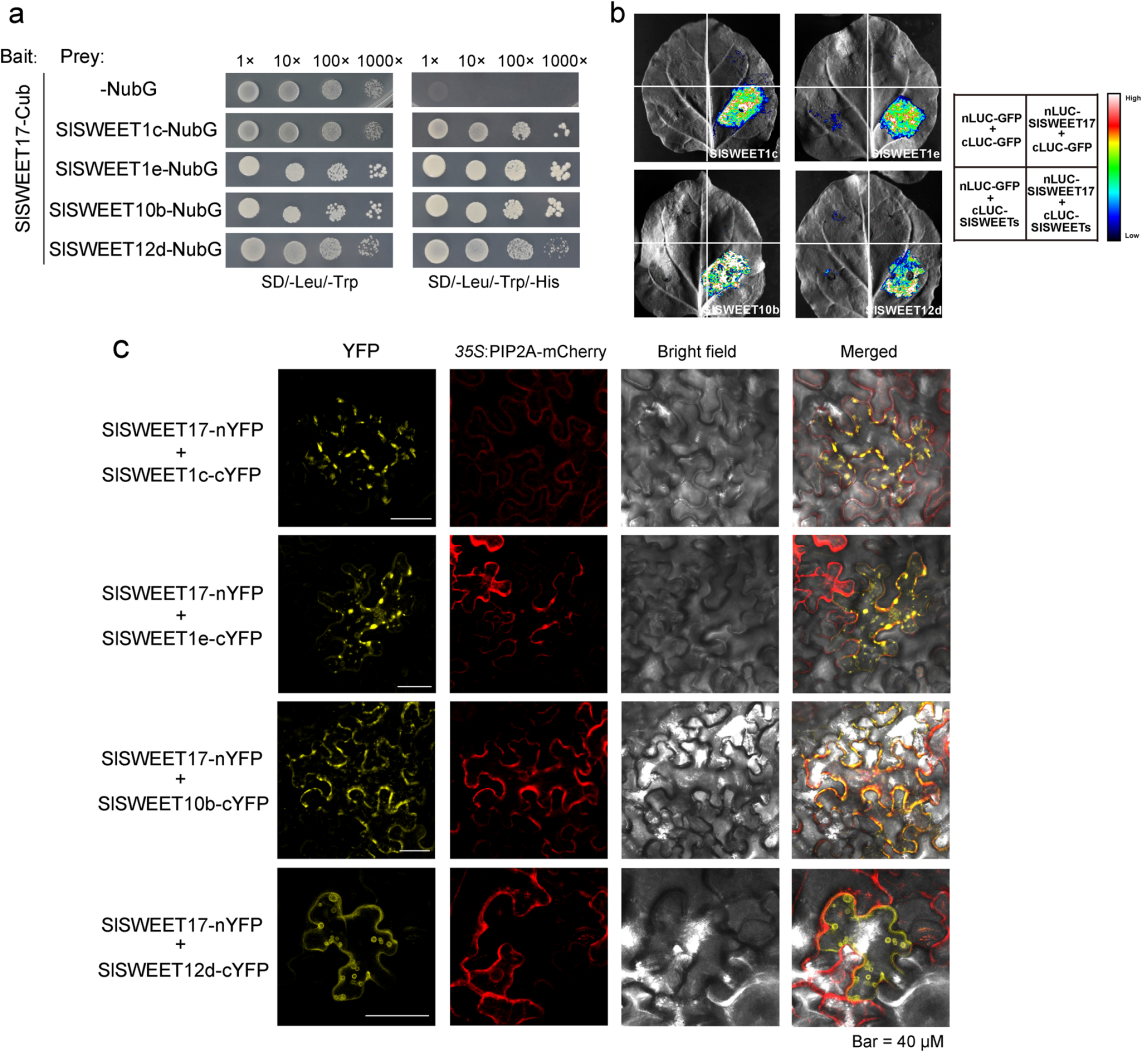
SlSWEET17 interacts with gall highly expressed SlSWEETs. a) Split ubiquitin membrane yeast two‐hybrid assays for interactions between SlSWEET17 and other SlSWEETs. The bait constructs contain the Cub fusions to SlSWEET17 and the prey constructs encode the SlSWEET1c, SlSWEET1e, SlSWEET10b or SlSWEET12d fused to the C terminus of NubG. The vectors were transformed into the yeast strain NMY51, and positive clones were spotted as 10‐fold serial dilutions on synthetic dropout (SD) medium lacking Leu and Trp, to confirm the presence of both plasmids. The synthetic dropout medium lacking Leu, Trp and His was used to screen for positive interactions. b) SlSWEET17 is associated with other SlSWEETs, as determined by split luciferase complementation assays. *Nicotiana benthamiana* leaves were transformed by injecting *Agrobacterium* GV3101 cells harbouring nLUC‐SlSWEET17 and cLUC‐SlSWEETs plasmids. nLUC‐GFP and cLUC‐GFP plasmids to serve as negative controls. Strong luciferase complementation was observed for the cLUC‐SlSWEETs and nLUC‐SlSWEET17 combinations, whereas no obvious signal was observed for the negative controls. All experiments were repeated at least three times with similar results. c) A bimolecular fluorescence complementation assay was used to demonstrate interaction in vivo. The nYFP‐SlSWEET17 and cYFP‐SlSWEETs constructs were cotransformed into *Nicotiana benthamiana* leaves. Bars = 40 µm. (SWEET, sugars will eventually be exported transporters; Cub, C‐ubiquitin; NubG, N‐ubiquitin which instead has a glycine at amino acid position 13; SD, synthetic dropout; LUC, luciferase; YFP, yellow fluorescence protein).

SWEET proteins normally form homo‐ or hetero‐oligomers to transport sugars.^[^ ^8^ ^]^ According to the RNA‐Seq data (Figure S2, Supporting Information), we selected the four most highly expressed *SWEETs* (*SlSWEET1c*, *SlSWEET1e*, *SlSWEET10b* and *SlSWEET12d*) for further study. Next, we examined the subcellular localization of these SWEET proteins. In tobacco leaves, these SlSWEETs were all found to localize in the plasma membrane (Figure S3, Supporting Information). In addition, co‐localization analysis showed that SlSWEET17 had co‐localization with other SlSWEETs on the plasma membrane, and SlSWEET1c and SlSWEET12d were also detected in Golgi bodies (Figure S4, Supporting Information). To determine whether SlSWEET17 functions synergistically with other SWEETs, we validated the interaction between the SlSWEET17 protein and the highly expressed SWEETs through the split‐ubiquitin membrane yeast two‐hybrid (MYTH) system. The results indicated that SlSWEET17 interacted with SlSWEET1c, SlSWEET1e, SlSWEET10b and SlSWEET12d (**Figure** **2**a). These interactions were also tested using a split‐luciferase system assay, which showed that SlSWEET17 formed hetero‐oligomers with the four SlSWEETs *in planta* (Figure 2b). Additionally, all of these interactions seemed to occur at the Golgi bodies, small vacuoles and plasma membrane according to the bimolecular fluorescent complimentary assay (BiFC), consistent with the subcellular localization of SlSWEET17 (Figure 2c; Figure S5, Supporting Information).

### 2.3 Reduced Sugar Uptake by SlSWEET17 Heterooligomers

**Figure 3:**
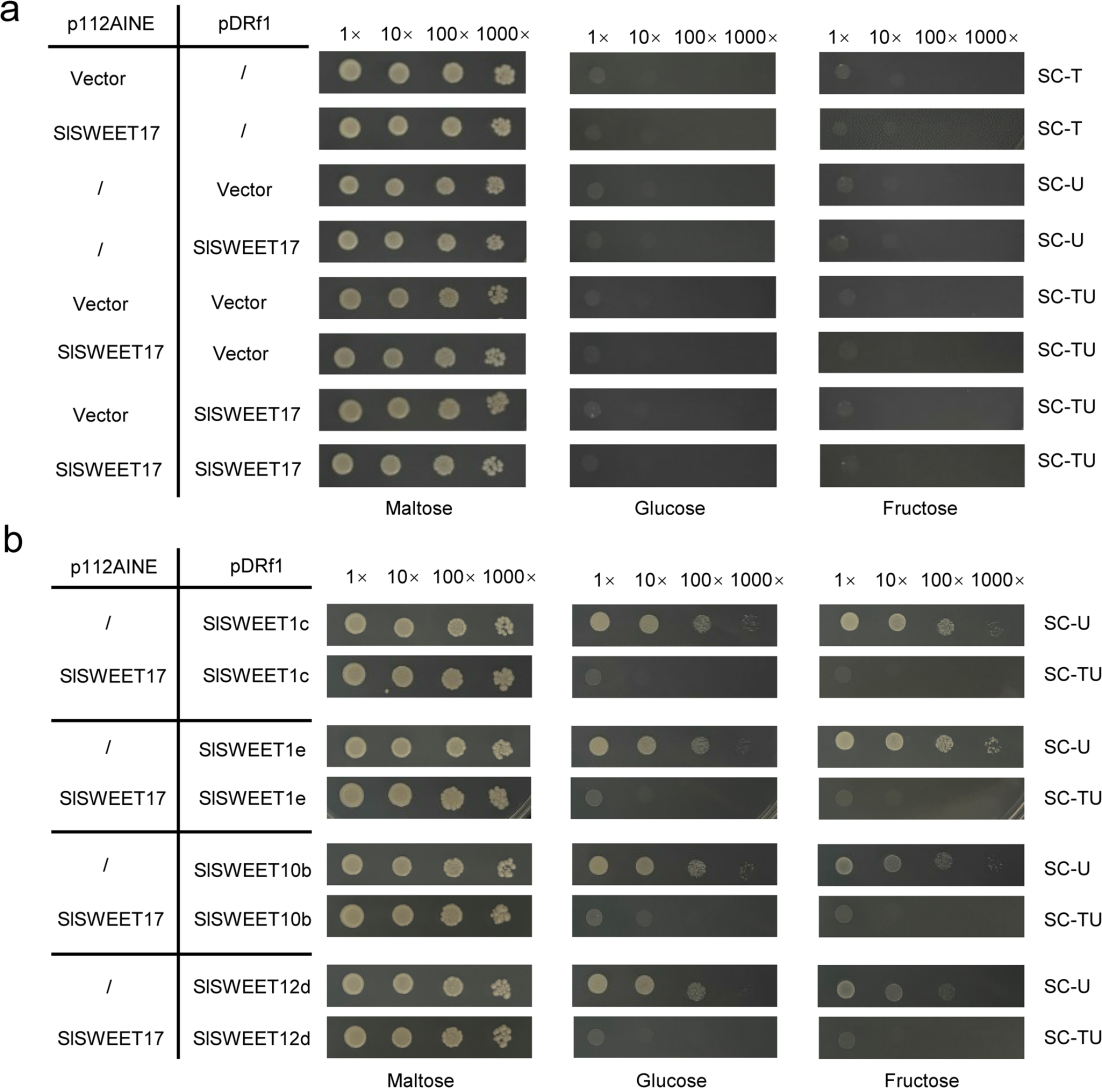
Impact on sugar transport by co‐expressing SlSWEET17 and other SlSWEET proteins in yeast strain EBY.VW4000. a) SlSWEET17 proteins were expressed from the strong ADH promoter (p112AINE‐SlSWEET17 vector) and strong PMA1 promoter (pDRf1‐SlSWEET17 vector), then transformed or co‐transformed into yeast hexose transporter mutant strain EBY.VW4000, respectively. The p112AINE empty vector and pDRf1 empty vector were used as negative control. SC selective mediums (‐Trp, ‐Ura or ‐Ura‐Trp) containing 2% maltose, glucose, or fructose were used for yeast growth assays. b) Growth assays of other SlSWEETs proteins alone or co‐transformed with SlSWEET17 in EBY.VW4000 yeast strain. SlSWEETs proteins were expressed from PMA1 promoter (pDRf1‐SlSWEETs) and was transformed or co‐transformed with pDR‐SlSWEETs vector into yeast hexose transporter mutant strain EBY.VW4000. Growth assays of yeast cells coexpressing SlSWEET17 and other SlSWEETs proteins were performed in SC selective medium (‐Ura or ‐Ura‐Trp) containing 2% maltose, glucose, or fructose. (SWEET, sugars will eventually be exported transporters; AHD, alcohol dehydrogenase; PMA, plasma membrane ATPase; T, Tryptophan; U, uracil; SC, synthetic complete).

To test the sugar transport activity of these SlSWEETs, the open reading frames (ORFs) of six SlSWEETs were amplified and cloned into a destination vector for protein expression in EBY.VW4000 yeast cells. Interestingly, we did not observe any glucose or fructose transport uptake of SlSWEET17 in the yeast expression systems (**Figure** **3**a). However, the remaining SlSWEETs could transport to transport glucose and/or fructose (Figure 3b). To further examine the function of oligomers, SlSWEET17 was co‐expressed with other SlSWEET proteins in yeast. SlSWEET17 was strongly expressed from the ADH promoter, whereas the other SlSWEETs were strongly expressed from the PMA1 promoter. Analysis of yeast colony growth on medium supplemented with maltose, glucose or fructose as the sole carbon source revealed that SlSWEET17 dramatically inhibited the glucose and/or fructose transport activity of SlSWEET1c, SlSWEET1e, SlSWEET10b and SlSWEET12d (Figure 3b). These results suggest that SlSWEET17 inhibits the sugar transport capacity of other SlSWEETs by forming heterooligomers.

### 2.4 SlSWEET17 Negatively Regulates Sugar Content in Galls and Susceptibility to RKNs

**Figure 4:**
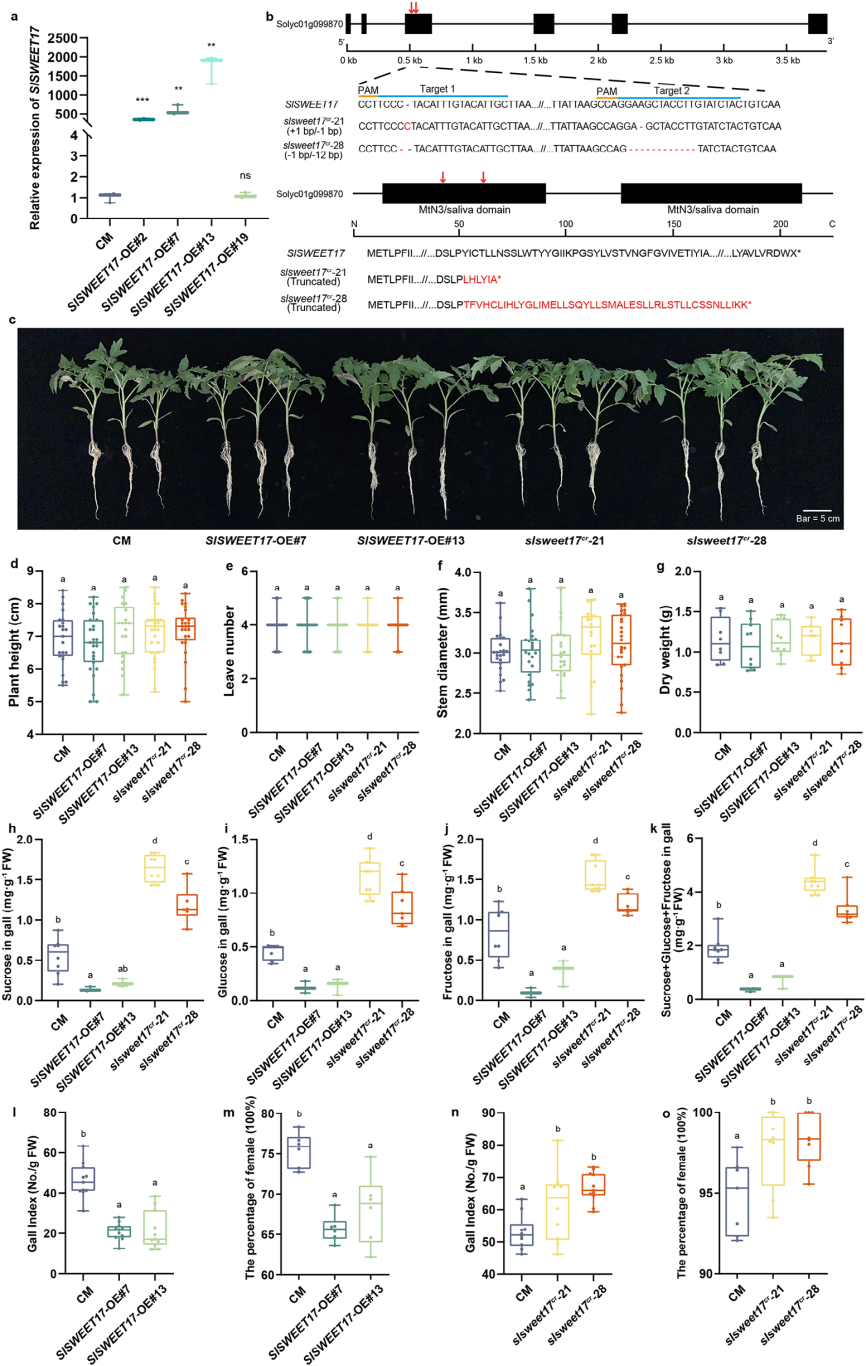
SlSWEET17 negatively regulated sugar content in galls and susceptibility to RKNs. a) The relative expression level of *SlSWEET17* in leaves of *SlSWEET17*‐OE and CM plants. Values are the mean ±SE of three biological replicates. Asterisk represents significant differences (**, *P* < 0.01; ***, *P* < 0.001). b) CRISPR‐Cas9 strategy for generating *SlSWEET17* loss‐of‐function plants. Target and PAM sequences are lined with blue and orange, respectively. Red letters indicate position of targets. c) The phenotype of different lines under normal growing environment. Plant height d), the number of leaves e), stem diameter f) and dry weight g) of different lines in normal growing environment (n ≥7). h–k) The sugar content (sucrose, glucose, fructose) in galls at 14 DPI (n = 6‐8). l, n) Gall indexes of CM, *SlSWEET17*‐OE and *slsweet17^cr^* plants at 14 DPI (n = 10‐11). m,o) The percentage of female of CM, *SlSWEET17*‐OE and *slsweet17^cr^* plants at 21 DPI (n = 6‐8). Different letters indicate significant differences by one‐way ANOVA using Tukey's multiple comparisons test. (SWEET, sugars will eventually be exported transporters; OE, overexpression; CM, tomato cultivar Castlemart; cr, CRISPR/Cas9 knockout; ns, non‐significant; PAM, protospacer adjacent motif; DPI, day post inoculation).

To test the role of SlSWEET17 in RKN infection, we overexpressed *SlSWEET17* driven by the *Super* promoter in the CM (Castlemart) tomato background. Positive transformants were characterized via qRT‐PCR (**Figure** **4**a), with *SlSWEET17*‐OE#7 and #13 selected for subsequent experiments. We then designed two target sites in the coding region of *SlSWEET17* using CRISPR (Clustered Regularly Interspaced Short Palindromic Repeats)/CRISPR‐associated protein 9 (Cas9) technology and generated two loss‐of‐function mutant alleles, *slsweet17^cr^‐*21 and *slsweet17^cr^‐*28. Sequencing revealed *slsweet17^cr^‐*21 mutant had single‐base insertion and single‐base deletion, and *slsweet17^cr^‐*28 contained single‐base deletion and small fragment deletions, both causing premature termination due to reading frame shift (Figure 4b). Under normal growth conditions, phenotypic analysis showed that *SlSWEET17* overexpression or knockout did not significantly affect tomato seedling development (Figure 4c–g). Given RKNs rely on host sugars for nutrition and soluble sugars (sucrose, glucose, fructose) dominate plant metabolism,^[^ ^17^ ^]^ we measured sugar levels in 14 DPI galls. Compared to CM plants, the galls from the *SlSWEET17*‐OE had reduced glucose, fructose, sucrose, and total sugar contents, whereas these contents were significantly increased in the *slsweet17^cr^* mutants (Figure 4h–k). Consistent with sugar content in galls, *SlSWEET17* overexpression caused significant reductions in gall index values and the percentage of females (Figure 4i,m), while mutations in the *SlSWEET17* gene resulted in markedly higher gall index values and percentage of females (Figure 4n,o). Interestingly, qRT‐PCR experiments showed that expression of the four SlSWEET genes decreased in SlSWEET17‐OE plants and increased in the *slsweet17^cr^* mutants (Figure S6, Supporting Information). These finding indicate SlSWEET17 negatively regulates soluble sugars accumulation via other SWEETs in galls during RKN infection, thereby reducing tomato susceptibility to RKN.

### 2.5 SlDOF9 Directly Negatively Regulates SlSWEET17 Expression

**Figure 5:**
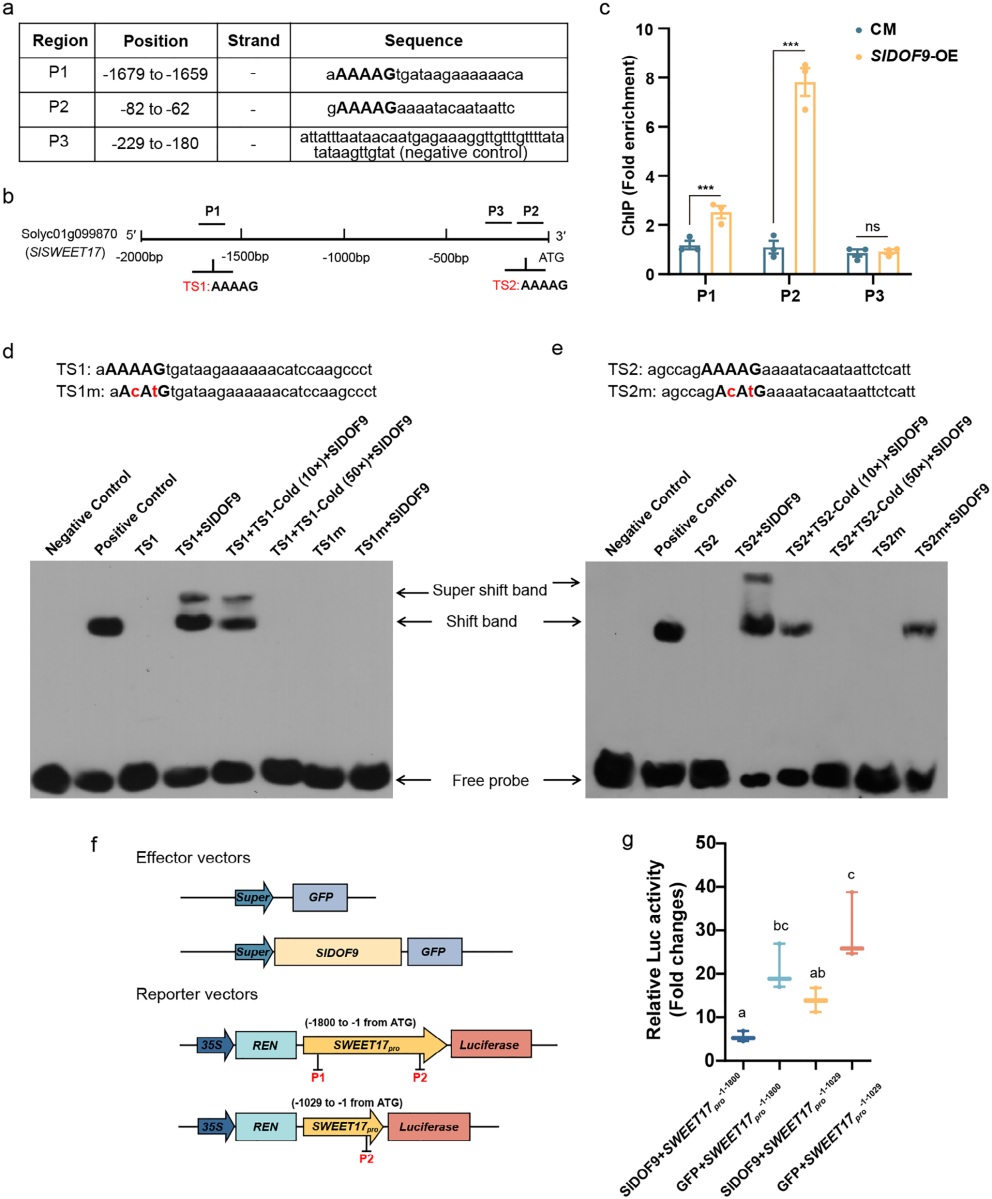
SlDOF9 directly binds the *SlSWEET17* gene promoter. a) Predicted binding sequence (P1 and P2) of SlDOF9 at the *SlSWEET17* promoter and P3 was a negative control sequence without any DOF binding motif. b) Schematic diagram of position of predicted binding sequences on the *SlSWEET17* promoter. c) SlDOF9 binds to P1 and P2 from the *SlSWEET17* promoter. Error bars indicate the ±SE (n = 3), and asterisks (***P < 0.001) indicate significant differences as determined by ANOVA followed by Dunnett's multiple comparisons test. d,e) EMSA showed SlDOF9‐binding activity to DNA fragments containing the potential target sites (TS1 and TS2) from *SlSWEET17* promoter, and mutation abolished or reduced the binding activity. m, mutant, indicated in red. The probe sequence is shown in Table S5 (Supporting Information). The positive control was performed with SlRR26‐His protein and SlPP2C probe. The negative control was performed with MBP‐His and SlSWEET17 labeled probe. f) Diagram of the Dual‐Luc reporter assay. g) The results of the Dual‐Luc reporter assay. The expression levels of LUC driven by the *SlSWEET17* promoter fragments of different lengths with or without SlDOF9. Error bars indicate the ±SE (n = 3). Different letters indicate significant differences by one‐way ANOVA using Tukey's multiple comparisons test. (SWEET, sugars will eventually be exported transporters; CM, tomato cultivar Castlemart; DOF, DNA binding with one finger; EMSA, electrophoretic mobility shift assay; TS, target site; TSm, mutated target site; GFP, green fluorescence protein; LUC, luciferase; REN, renilla luciferase).

To identify upstream transcription factors of *SlSWEET17*, its promoter was analyzed using PlantPAN 4.0 (web‐based tool for analyzing promoters) (Table S2, Supporting Information). DOF transcription factor‐binding sites were the most abundant (Table S3, Supporting Information). Among the DOF transcription factors, the binding sites of AtDOF3.4 and AtDOF5.8 were maximum. And *SlDOF9*, a homologous gene of *AtDOF3.4* and *AtDOF5.8*, was predicted to bind with the target sequences (TSs) a**AAAAG**tgataagaaaaaaca and aaagccag**AAAAG**aaaatacaataattct including the core motif (**AAAAG**) (**Figures** **5**a and S4, Supporting Information). The position of the identified TS in the *SlSWEET17* promoter was shown in Figure 5b. Subcellular localization analysis revealed SlDOF9 localized to the nucleus, confirming its transcription factor identity (Figure S7a, Supporting Information). Based on predicted TS, we designed corresponding primers to detect SlDOF9 binding fragment by chromatin immunoprecipitation (ChIP)‐qPCR. Using GFP‐tagged *SlDOF9*‐OE plants, ChIP‐qPCR showed that SlDOF9 bound P1 and P2 regions rather than the P3 region (negative control) of the *SlSWEET17* promoter (Figure 5c). Furthermore, to verify the core motif within the target sites, a super‐shift electrophoretic mobility shift assay (EMSA) was used. SlDOF9 could bind to TS1 and TS2 in the promoter of SlSWEET17 in vitro, and these interactions were blocked by mutations in the core motif of TS1, but mutations at two bases were not sufficient to prevent SlDOF9 from binding at the TS2 site (Figure 5d,e). Moreover, Dual‐luciferase (Dual‐Luc) reporter assay was performed and verified that co‐expression of the *Super*:SlDOF9 and *SlSWEET17pro*:LUC constructs significantly decreased the luminescence intensity relative to that of the controls (GFP + *SlSWEET17_pro_*) (Figure 5f,g). In addition, qRT‐PCR analysis in galls showed that *SlDOF9* expression declined in the early stage of RKN infection (Figure S7b, Supporting Information), which was opposite to the expression trend of *SlSWEET17*. Taken together, these results demonstrate that SlDOF9 directly inhibit the expression of *SlSWEET17*.

### 2.6 SlDOF9 Positively Regulates Sugar Content in Galls and Susceptibility to RKNs

**Figure 6:**
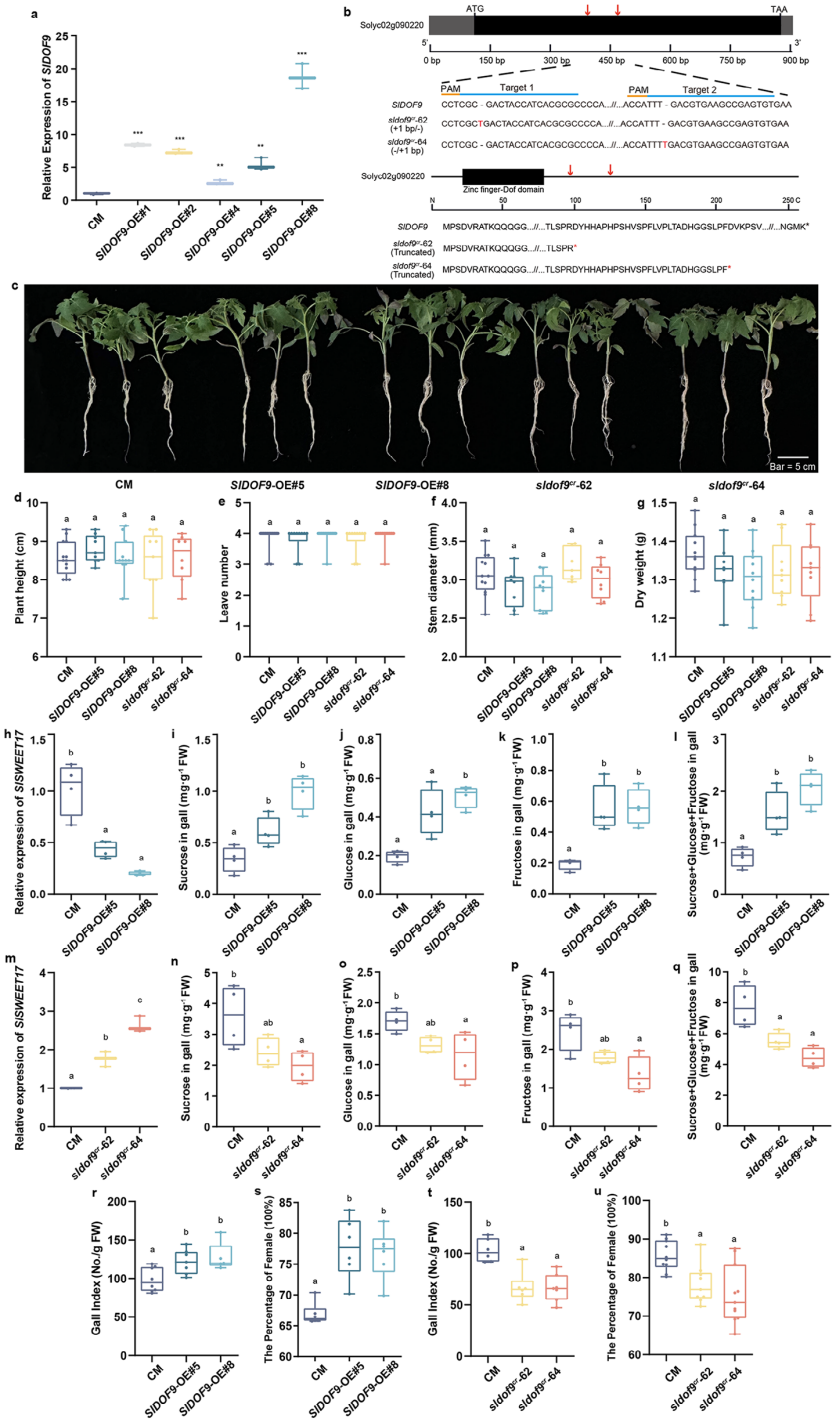
SlDOF9 positively regulated sugar content in galls and susceptibility to RKNs. a) The relative expression of *SlDOF9* in leaves of *SlDOF9*‐OE and CM plants. Error bars indicate the ±SE (n = 4). Asterisk represents significant differences (***, *P* < 0.001). ns, non‐significant. b) CRISPR‐Cas9 strategy for generating *SlDOF9* loss‐of‐function plants. Target and PAM (protospacer adjacent motif) sequences are lined with blue and orange, respectively. Red letters indicate position of two targets. c) The phenotype of different lines under normal growing environment. Plant height d), the number of leaves e), stem diameter f) and dry weight g) of different lines in normal growing environment (n > 7). h,m) The expression of *SlSWEET17* in galls of *SlDOF9*‐OE, *sldof9^cr^* and CM plants at 14 DPI (day post inoculation). Error bars indicate ±SE (n = 4). i–l) The sugar content (sucrose, glucose, fructose) in galls of *SlDOF9*‐OE, *sldof9^cr^* and CM plants at 14 DPI (n = 4). n–q) Gall indexes of *SlDOF9*‐OE, *sldof9^cr^* and CM plants at 14 DPI (n = 6‐8). r‐u) The percentage of female of *SlDOF9*‐OE, *sldof9^cr^* and CM plants at 21 DPI (n = 6‐10). Different letters indicate significant differences as determined by ANOVA followed by Tukey's multiple range test. All experiments were repeated at least three times. (CM, tomato cultivar Castlemart; OE, overexpression; cr, CRISPR/Cas9 knockout; PAM, protospacer adjacent motif, DPI, day post inoculation).

SlDOF9 has been reported to regulate inflorescence and flower development in tomato.^[^ ^18^ ^]^ However, whether SlDOF9 is involved in tomato‐RKN interactions is unclear. To test the role of SlDOF9 in planta, SlDOF9 was overexpressed in the CM genetic background, and the *SlDOF9*‐OE#5 and *SlDOF9*‐OE#8 lines were selected for subsequent experiments (**Figure** **6**a). We designed two target sites in the coding region of *SlDOF9* using Cas9 technology and generated two loss‐of‐function mutant alleles, *sldof9^cr^‐*62 and *sldof9^cr^‐*64. Both *sldof9^cr^* mutants had a single‐base insertion, which led to premature termination of translation after the reading frame shift (Figure 6b). Then, we observed the phenotypes of different lines under normal growth conditions. Similar to the situation of *SlSWEET17*, overexpression or knockout of *SlDOF9* did not affect tomato seedling development (Figure 6c–g). We analyzed *SlSWEET17* expression in the galls of *SlDOF9*‐OE and *sldof9^cr^* lines at 14 DPI. The results showed that the transcription of *SlSWEET17* decreased in *SlDOF9*‐OE plants but increased in *sldof9^cr^* mutants (Figure 6h,m). Next, we determined the soluble sugar content in galls. Compared to CM, the sucrose, glucose, and fructose contents and the sum of all three significantly increased in *SlDOF9*‐OE plants and decreased in *sldof9^cr^* mutants (Figure 6i–l,n–q), which was in contrast to the pattern of *SlSWEET17*. Furthermore, the analysis of gall index values and percentage of females showed that the *SlDOF9*‐OE lines were more susceptible to RKN than CM, whereas the *sldof9^cr^* mutants were less susceptible (Figure 6r–u). These results suggest that SlDOF9 positively regulates the sugar content in galls and the susceptibility of tomato plants during RKN infection.

### 2.7 SlDOF9‐Mediated Tomato Susceptibility to RKN Dependent on SlSWEET17

**Figure 7:**
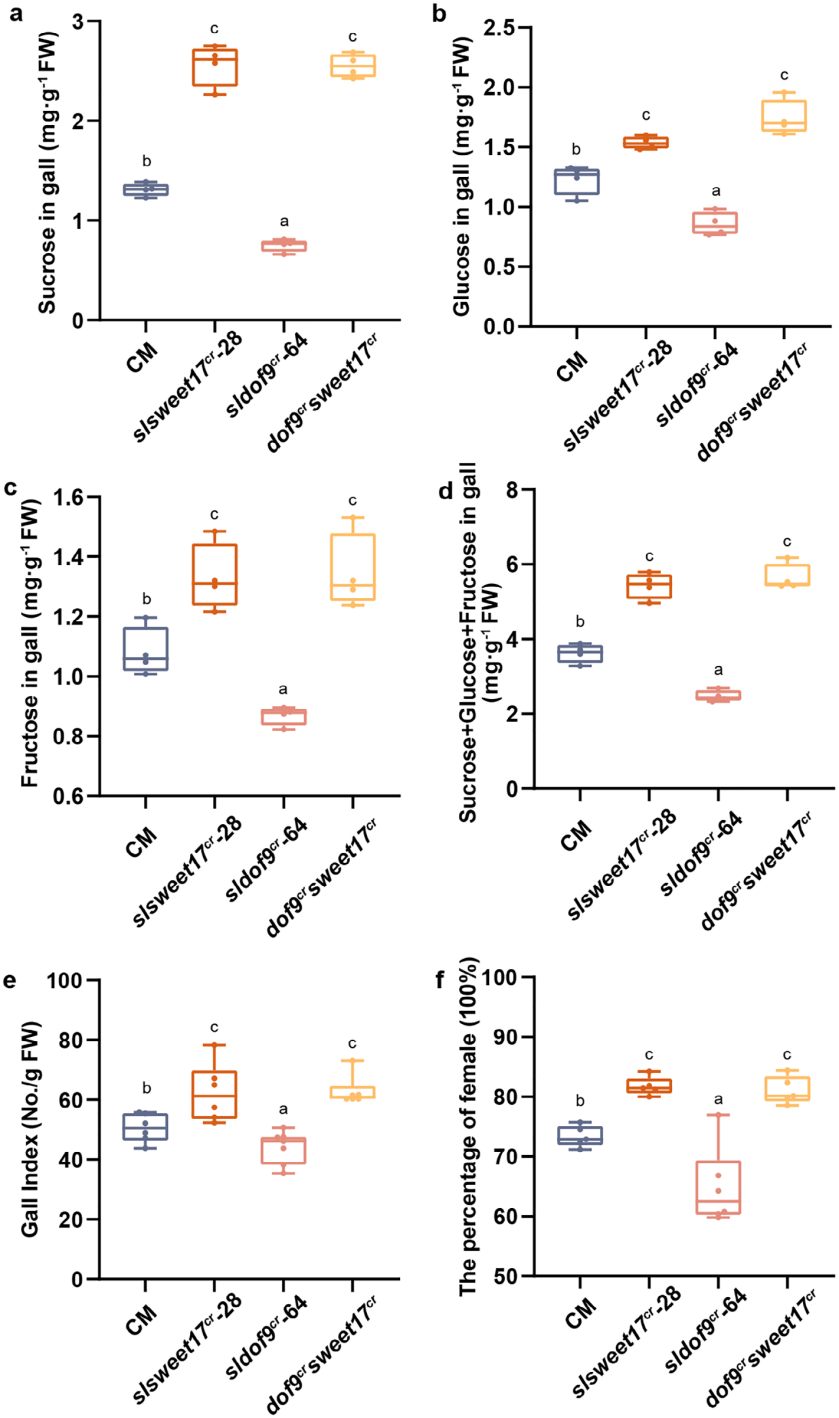
The regulation of SlDOF9 on plant susceptibility to RKNs and sugar in galls dependent on SlSWEET17. a–d) The sugar content (sucrose, glucose, fructose) in galls of *slsweet17^cr^*, *sldof9^cr^*, *dof9^cr^sweet17^cr^* and CM plants at 14 DPI (n = 4). e) Gall indexes of *slsweet17^cr^*, *sldof9^cr^*, *dof9^cr^sweet17^cr^* and CM plants at 14 DPI (day post inoculation) (n = 7‐8). f) Female proportion of *slsweet17^cr^*, *sldof9^cr^*, *dof9^cr^sweet17^cr^* and CM plants at 21 DPI (n = 6). Different letters indicate significant differences as determined by ANOVA followed by Tukey's multiple range test. All experiments were repeated at least three times. (CM, tomato cultivar Castlemart; SWEET, sugars will eventually be exported transporters; DOF, DNA binding with one finger; OE, overexpression; cr, CRISPR/Cas9 knockout; DPI, day post inoculation).

To further clarify the regulatory relationship between SlDOF9 and SlSWEET17, we generated *dof9^cr^sweet17^cr^* double mutants by crossing *sldof9^cr^* with *slsweet17^cr^* (Figure S8, Supporting Information). Sucrose, glucose, and fructose and the sum of these three sugars were higher in *dof9^cr^sweet17^cr^* galls than in CM. However, no significant differences in sugar content were detected between the *dof9^cr^sweet17^cr^* and *slsweet17^cr^* mutants (**Figure** **7**a–d). Similar, the gall index values and the percentage of females increased in the *dof9^cr^sweet17^cr^* mutants relative to CM plants, whereas no obvious difference was detected in the *slsweet17^cr^* group (Figure 7e,f). These results suggested that SlDOF9‐mediated regulation of plant susceptibility to RKNs is dependent on SlSWEET17.

## 3 Discussion

SWEET transporters are pH‐independent bidirectional sugar transporters that mediate concentration gradient‐driven diffusion, and they are also candidate proteins that can be hijacked by microbes to intercept sugars. RKN parasitism requires the acquisition of photosynthetic output from the host. As feeding sites, GCs are mainly regulated by RKNs and consume nutrients from surrounding tissues. In this study, however, we found that the SlDOF9‐SlSWEET17 regulatory model is a strategy for plants to actively fight RKN. And we show that SlSWEET17, as a negative regulator of the SWEET family, affects sugar transport and host susceptibility to RKN by inhibiting the function of other SWEET proteins.

Research on the SWEET sugar transporters over the past decade has shown that SWEETs are involved in important physiological processes due to their sugar transport ability.^[^ ^19^ ^]^ For instance, *AtSWEET11* and *AtSWEET12* mediate sucrose efflux from putative phloem parenchyma into the phloem apoplast, a key step in phloem loading.^[^ ^7b^ ^]^ *AtSWEET8* plays a role in glucose efflux from the tapetum to support pollen growth and affects male fertility.^[^ ^20^ ^]^ *AtSWEET5/VEX1* (*Vegetative Cell Expressed 1*), which encodes a glucose transporter, provides sugars to support germ cell development within pollen grains.^[^ ^21^ ^]^ *Petunia hybrida* NEC1 (a homolog of *AtSWEET9*) is involved in nectar secretion.^[^ ^22^ ^]^ *AtSWEET17*, expressed in the parenchyma and vascular tissue, functions as a fructose‐specific uniporter in the tonoplast, exporting fructose from vacuoles and controlling the fructose content in leaves.^[^ ^23^ ^]^ However, SlSWEET17, in contrast to its fructose‐specific homolog AtSWEET17, does not seem to have glucose or fructose transport capacity in yeast (Figure 3a). SlSWEET17 localized to the plasma membrane, small vacuoles and Golgi bodies (Figure 1c), implying that its function is different from that of *AtSWEET17*.

Several studies have shown that mutualistic microbes hijack specific sugar transporters to import rhizospheric sugars produced by plants.^[^ ^6^, ^9^, ^10^ ^]^ In our research, RKN infection significantly induced SlSWEET17 expression (Figure 1a,b), but *SlSWEET17* overexpression decreased the gall index and female proportion of RKN (Figure 4l,m), suggesting that SlSWEET17 negatively regulates the sensitivity of the host to RKNs in tomato. More interestingly, the yeast sugar absorption results showed that SlSWEET17 had no ability to absorb glucose or fructose (Figure 3a), suggesting that SlSWEET17 does not regulate sugar distribution through its own transport capacity. Previous studies have shown that SWEETs require homo‐ and hetero‐oligomerization for the sugar transport capacity.^[^ ^8^, ^24^ ^]^ Thus, we attempted to clarify the sugar transport capacity of hetero‐oligomers formed with SlSWEET17. Our results suggest that the sugar transport capacity of most other SlSWEETs was significantly disrupted by oligomerization with SlSWEET17 in yeast (Figure 3b), which is consistent with the decrease in sugar content observed in the galls of *SlSWEET17*‐OE plants and the increase in sugar content observed in the galls of *slsweet17^cr^* mutants (Figure 6h–k). Combined with the subcellular localization and BiFC assay, we hypothesize that the subcellular localization of other SlSWEET proteins may be altered by interactions with SlSWEET17 (Figure 2; Figure S3, Supporting Information), which may also result in alterations of their sugar transport activity. Furthermore, the interaction between SlSWEET17 and other SlSWEETs may affect the posttranslational modifications of these SlSWEET proteins, such as phosphorylation, which has also been reported to be involved in regulation of the transport capacity of SWEET proteins.^[^ ^25^ ^]^ In addition, some amino acid residues play important roles in the formation of functional transport channels in SWEETs,^[^ ^26^ ^]^ and the binding of SlSWEET17 to other SlSWEETs might affect this process and inhibit sugar efflux.

Previous studies have shown that bacterial pathogens, such as blight bacteria (*Xanthomonas oryzae*), secrete transcription activation‐like effector proteins (TALEs) to activate the *OsSWEET* promoter in rice to obtain sugar.^[^ ^7^, ^9^, ^27^ ^]^ Recently, it was reported that the expression of 17 tomato *SlSWEETs*, including SlSWEET17, was induced in roots after RKN inoculation.^[^ ^28^ ^]^ TALE binding sites in the promoter of *OsSWEET13* edited by CRISPR/Cas9 technology produced germplasm resistant to multiple leaf blight bacterial isolates.^[^ ^9d^ ^]^ However, no homologous protein of TALEs has been found in the RKN genome. This finding suggested an unknown mechanism for activation of SWEETs transcription. Here, we found that SlDOF9 transcription factor was downregulated in early stage of response to RKN inoculation (Figure S6b, Supporting Information) and could directly bind to the *SlSWEET17* promoter and negatively regulate the expression of the *SlSWEET17* gene (Figure 5 and Figure 6h,m). DOF transcription factors regulate multiple progress of plant growth and development. In previous reports, SlDOF9 was involved in the development of tomato inflorescence mediated by auxin signaling, and the *sldof9‐KO* mutant promoted the number of flowers per inflorescence and increased fruit yield with vibration‐assisted flower pollination conditions.^[^ ^18^ ^]^ Combined with our study, *sldof9^cr^* mutants influenced the sugar transport by SlSWEET proteins, which might affect the accumulation of sugar in fruit, thus increasing tomato yield. And OsDOF11 can directly bind to the promoter of OsSUT1, OsSWEET11 and OsSWEET14, promote the expression of these genes, affect the growth and development of rice by regulating sucrose transport, and affect the susceptibility of rice to *Xanthomonas oryzae* pathovar *oryzae* by regulating the expression of OsSWEET11.^[^ ^14a^ ^]^ These results suggests that DOF transcription factors play an important role in the regulation of sugar transport.

Our study revealed the dynamic regulation of sugars by the SlDOF9‐SlSWEET17 module in RKN infection, which promoted the exploration of the mechanism of plant‐pathogen interaction centered on nutrient distribution, and proposed the scheme of accurate regulation of sugar transport pathway by the protein interaction of SWEET family, providing a critical genetic target for breeding crops with reduced susceptibility to RKNs.

Meanwhile, this study also had several limitations. First, the molecular basis of the interaction between SlSWEET17 and other SlSWEETs remained unclear. Although we proposed that SlSWEET17 inhibits the functions of other SlSWEETs by altering their subcellular localization, there was not clear how SlSWEET17 leads to the suppression of other SWEET transporters’ activity. Secondly, we focused on the relationship between SlSWEET17 and the SWEET family members highly expressed in the galls, and it was unclear whether this inhibition of SlSWEET17 extends to all members of the SWEET family or in other plant organs. Our next research direction will be to better understand the nutrient competition network between plants and pathogens to provide theoretical basis for the creation of disease‐resistant materials.

**Figure 8:**
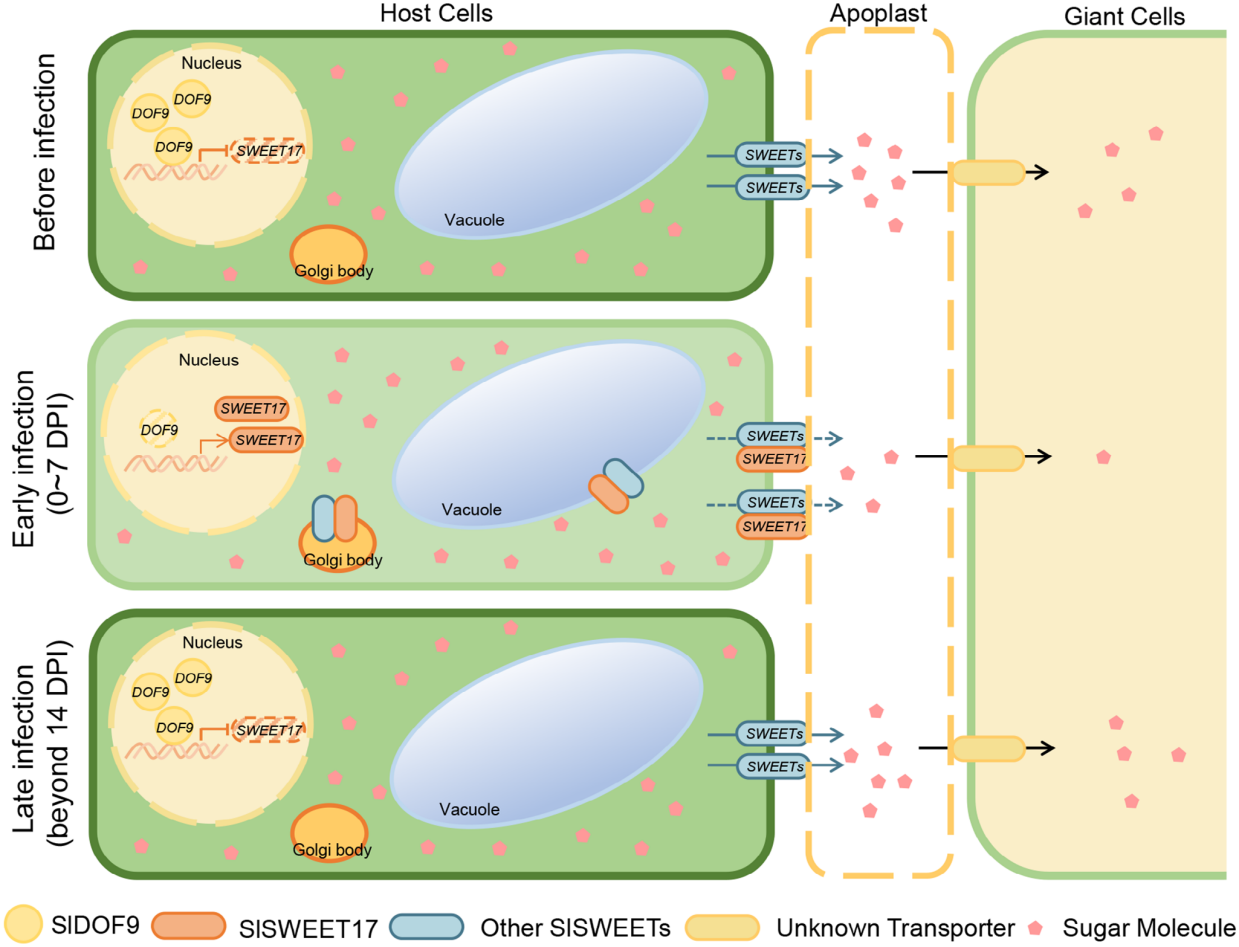
A proposed model for SlDOF9‐SlSWEET17 module mediated sugar distribution in roots and galls. There are three stages of SlDOF9‐SlSWEET17 module to regulate sugar distribution between roots and galls. Before the infection by RKNs, *SlSWEET17* and *SlDOF9* were expressed at default levels, ensuring that other SlSWEET proteins could transport sugar into the apoplast. During the early stage of infection (0–7 DPI), the expression of *SlDOF9* is downregulated, and the inhibition of *SlSWEET17* expression by SlDOF9 is alleviated. More SlSWEET17 proteins are translated and interact with other SlSWEET proteins, which leads to changes in the spatial position of plasma membrane‐localized SlSWEET proteins and a decrease in the sugar transport capacity of these SlSWEETs. In the later stage of RKNs infection (beyond 14 DPI), SlDOF9 is up‐regulated and SlSWEET17 is suppressed, the other SlSWEET proteins are hijacked by RKNs, promoting the transport of sugar into apoplast and providing essential nutrients for the GCs. (SWEET, sugars will eventually be exported transporters; DOF, DNA binding with one finger; DPI, day post inoculation; GCs, giant cells).

In summary, we propose a model to describe how SlDOF9‐SlSWEET17 model acts as a switch to regulate sugar distribution between host and galls, which consists of three phases (**Figure** **8**). Before the infection by RKNs, *SlSWEET17* and *SlDOF9* were expressed at default levels, ensuring that other SlSWEET proteins could transport sugar into the apoplast. During the early stage of infection (0–7 DPI), the expression of *SlDOF9* is downregulated (Figure S6b, Supporting Information), relieving the SlDOF9‐mediated inhibition of *SlSWEET17* (Figure 1b). Subsequently, SlSWEET17 protein is translated and transported to the Golgi (Figure 1c), followed by vesicle transport to different subcellular compartments. Some of these SlSWEET17 molecules appear to interact with other SlSWEETs in the Golgi body (Figure 2c), which likely affects the location or sugar transport activity of other SlSWEETs, less sugar in cells is exported to the apoplast by other SlSWEETs, which means that even less sugar is transported into the GCs by unknown sugar transporters. This is likely an immune response elicited by the host plant to suppress RKN infection. In the later stage of RKNs infection, SlDOF9 is up‐regulated and SlSWEET17 is suppressed (Figure 1b; Figure S6b, Supporting Information), the other SlSWEET proteins are again hijacked by RKNs, promoting the transport of sugar into apoplast and providing essential nutrients for the GCs. This may be elicited by RKN, possibly through effectors, to facilitate the infection process. Overall, SlDOF9‐SlSWEET17 model helps plants resist RKN infection during early stage by switching off the sugar transport capacity of other SlSWEET proteins that are hijacked by RKNs.

## 4 Conclusion

The SlDOF9‐SlSWEET17 regulatory module acts as a dynamic switch controlling sugar partitioning between roots and galls during RKN infection. This mechanism illustrates a critical plant‐pathogen tug‐of‐war, where regulation of sugar transport determines the nutrient balance between plants and parasites.

## 5 Experimental Section

### Plant Materials and Growth Conditions

The tomato (*Solanum lycopersicum* L.) cultivar Castlemart (CM) was used as the wild type (WT) in this study. Tomato seeds were surface‐sterilized with 2.5% (*v/v*) sodium hypochlorite solution for 15 min, washed at least five times with sterile water, sown on soil in a small pot and subsequently grown in a growth chamber under a 16:8 h light/dark photoperiod, with LED light at an intensity of 250 µmol m^−2^ s^−1^, 60% relative humidity and 24 °C light/18 °C darkness. All overexpression and mutant lines were constructed on the CM genetic background. *Nicotiana benthamiana* plants were cultivated in a growth chamber under a 16:8 h light/dark photoperiod with 22 °C light/18 °C darkness, 200 µmol m^−2^ s^−1^ light intensity and 70% relative humidity in a pot filled with a 2:1 mixture of peat and vermiculite.

### Nematode Culture and Inoculation

A population of the nematode *Meloidogyne incognita* was maintained on water spinach (*Ipomoea aquatica* Forssk.). Inoculation and susceptibility characterization were performed based on previously published studies.^[^ ^29^ ^]^ Notably, the gall index was calculated as the number of galls per gram of fresh root weight per plant at 14 DPI. To calculate the percentage of females, after 21 DPI, the tomato roots with galls were gently washed with distilled water, then frozen and thawed three times in a freezer at −20 °C. Put the roots with galls into a blender and blend for ≈8 s. The crushed material was filtered through a 200‐mesh nylon net, and washed into a 600‐mesh nylon net with uniform water flow. Then collected it into a 50 mL centrifuge tube and let it settle at the bottom. After that, removed the supernatant and pipetted the sediment into a 6‐well plate, counted the number of female nematodes under an optical microscope (SMZ‐140; Motic, Guangdong, China) according to previously described method.^[^ ^30^ ^]^

### Plant Transformation

To induce overexpression of *SlSWEET17* and *SlDOF9* with overexpression vectors, the coding sequences of *SlSWEET17* and *SlDOF9* coding sequences were amplified by PCR using the primers listed in Table S5 (Supporting Information), and the fragments were inserted into the pCAMBIA1300‐GFP vector harboring a Super enhancer promoter ^[^ ^31^ ^]^ fused with green fluorescent protein (GFP) to generate the *Super:SlSWEET17‐GFP* and *Super:SlDOF9‐GFP* vectors, respectively. These constructs were subsequently transformed into CM tomato plants via *Agrobacterium tumefaciens* (GV3101)‐mediated cotyledon explant transformation. T3 plants homozygous for the overexpression sequence were used for the experiments. To generate *sweet17^cr^* and *dof9^cr^* mutants with CRISPR/Cas9 technology, potential guide sequences were predicted by the website CRISPOR (http://crispor.tefor.net/) (Figures S6 and S7, Supporting Information). CRISPR/Cas9 mutagenesis was performed as previously described.^[^ ^32^ ^]^ CRISPR/Cas9‐induced mutations were genotyped by PCR amplification and DNA sequencing. Cas9‐free T2 homozygotes were selected for the experiments. The primers used for genotype identification were shown in Table S5 (Supporting Information).

### RNA Extraction and qRT‐PCR Analysis

At 0, 7, 14, 21 and 28 DPI, the tomato roots were gently washed out with distilled water. Then the galls and the part of the root that surrounded the galls (without root tips) were cut for RNA extraction. And uninfected roots were used as controls. Total RNA was extracted using Plant RNA Extract Kit (DP432, Tiangen, Beijing, China) according to the manufacturer's protocol, and first‐strand cDNAs were synthesized from 1 µg RNA using ReverTra Ace qPCR RT Master Mix with gDNA Remover (FSQ‐301, TOYOBO, Shanghai, China). qRT‐PCR analyses were conducted using the ABI (Applied Biosystems) Prism 7500 Fast real‐time PCR system (Thermo Fisher Scientific, Waltham, MA, USA). The *beta‐actin* gene (Solyc11g005330) was selected as a control. At least three biological replicates were included. The primers used for qRT‒PCR were listed in Table S5 (Supporting Information).

### Subcellular Localization Analysis

For the subcellular localization assay, the 1500‐bp of *SlSWEET17* promoter was cloned, and generated the recombinant *SWEET17_pro_:SlSWEET17‐GFP* construct. Both the *Super:SlSWEET17‐GFP* and *SWEET17_pro_:SlSWEET17‐GFP* vectors were transformed into hairy roots of tomato via *Agrobacterium rhizogenesis*‐mediated cotyledon transformation.^[^ ^33^ ^]^ The CDSs of other SWEETs were amplified by PCR using the primer pairs Super‐SWEET1c/SWEET1e /SWEET10b/SWEET12d/SWEET17‐GFP‐F/R and inserted into the *pCAMBIA1300‐Super:GFP* vector. The S*uper:SlSWEETs‐GFP* and *Super:SlDOF9‐GFP* vectors were introduced into *N. benthamiana* leaves. The GFP fluorescence of hairy roots and leaves was visualized with a confocal laser scanning microscope (Leica TCS SP5; Leica Camera AG, Wetzlar, Germany). For GFP imaging, a 488‐nm argon laser was used for excitation, and emission was detected at 505–530 nm.

The CDSs (Coding sequences) of the SWEETs were amplified by PCR using the primer pairs BP‐SWEET1c/SWEET1e/SWEET10b/SWEET12d/SWEET17‐GFP‐F/R (listed in Table S5, Supporting Information). Target PCR fragments were purified and inserted into the Gateway entry vector pDONR221‐f1 via a BP recombination reaction (recombination of attB sites with attP sites) (Invitrogen, Cat#11789020). Colony PCR and sequencing were used to identify plasmids with the correct sequences. Then, the entry clone plasmids were mixed with the destination vector pDRf1‐GFP‐GW for the LR recombination reaction to generate *PMA1:SWEETs‐GFP* vectors (via recombination of attL sites with attR sites) (Invitrogen, Cat#11791020). The yeast hexose transporter mutant strain EBY.VW4000 was used to express PMA1:SWEETs‐GFP fusion proteins. GFP fluorescence was detected by confocal laser scanning microscopy, and the excitation wavelengths and emission wavelengths were 488 nm and 505–530 nm, respectively.^[^ ^8^ ^]^

### Complementation Assays and Co‐Expression of Different SWEETs in the Yeast

The open reading frame of SWEETs was amplified by PCR using the primer pairs BP‐SWEET1c/SWEET1e/SWEET10b/SWEET12d/SWEET17‐F/R (listed in Table S5, Supporting Information) and cloned into the Gateway entry vector pDONR221‐f1 via the BP reaction. Then, the *SWEETs* gene was transferred into the destination vector pDRf1‐GW via the LR reaction, and *SWEET17* was also transferred into the destination vector p112AINE‐GW via the LR reaction. The pDRf1‐GW‐ and p112AINE‐GW‐SWEETs plasmids were transformed individually or together into the yeast hexose transporter mutant EBY.VW4000 via lithium acetate transformation. Transformants were first grown on synthetic complete (SC) selective medium (‐Ura) (for pDRf1‐GW‐SWEETs individual transformation) and SC selective medium (‐Ura and ‐Trp) (for pDRf1‐GW‐SWEETs and p112AINE‐GW‐SWEETs co‐transformation) supplemented with 2% (*v*/*v*) maltose (Sigma–Aldrich, St. Louis, MO, USA) as the sole carbon source at 30 °C for 4 days. The EBY.VW4000 cells expressing SWEETs proteins were grown in SC liquid selective medium (‐Ura or ‐Ura‐Trp) and resuspended in 0.9% (*w/v*) NaCl solution to achieve an OD_600nm_ (Optical Density in 600 nm) of ≈0.6. Serial dilutions of yeast cell suspensions were added dropwise onto solid SC selective medium (‐Ura or ‐Ura‐Trp) consisting of 2% maltose, 2% glucose, 2% fructose or 2% galactose. Growth was recorded by imaging after 3–4 days of growth at 30 °C.^[^ ^8^ ^]^

### Split‐Ubiquitin Membrane Yeast Two‐Hybrid Assays

The coding regions of *SlSWEET17* were amplified and cloned into a pBT3‐STE bait vector, which contain the Cub fusions, and the coding regions of *SlSWEET1c*, *SlSWEET1e*, *SlSWEET10b* and *SlSWEET12d* were amplified and cloned into a pPR3‐C prey vector, which contain the mutant variant of Nub (NubG). Then, the vectors were transformed into the yeast strain NMY51 according to the user manual (Dualsystems Biotech, Schlieren, Switzerland). Yeast transformation was performed with PEG/LiAc using a Yeastmaker Yeast Transformation System 2 Kit (Clontech, Takara, Japan). After positive clones were confirmed, toxicity and self‐activation assays of the bait plasmid were performed as previously described.^[^ ^24^ ^]^ All the assays were repeated at least three times.

### Bimolecular Fluorescence Complementation (BiFC) Assays

For BiFC assays, the CDS of *SWEET17* was inserted into the pCAMBIA1300‐nYFP vector, and the nYFP was expressed as a fusion protein at the N‐terminus of SWEET17. The CDSs of *SWEET1c*, *SWEET1e*, *SWEET10b* and *SWEET12d* were cloned into the pCAMBIA1300‐cYFP vector to express the corresponding cYFP‐SWEET fusion proteins. The methods used for co‐infiltration and observation were previously described.^[^ ^29^ ^]^

### Firefly Luciferase Complementation Assay

For the LCI assays, the CDS of *SWEET17* was cloned into the pCAMBIA‐nLUC vector, and the CDSs of *SWEET1c*, *SWEET1e*, *SWEET10b* and *SWEET12d* were cloned into the pCAMBIA‐cLUC vector. The recombinant vectors were co‐infiltrated into *Nicotiana benthamiana* leaves. Luciferase complementation images were obtained as previously described.^[^ ^29^ ^]^

### Dual‐Luc Assay

Two fragments (−1800 bp to −1 bp from ATG and‐1029 bp to −1 bp from ATG) of the *SlSWEET17* promoter were inserted into the pGreenII 0800‐LUC vector as reporters, and *Super:SlDOF9‐GFP* vectors were used to generate the SlDOF9‐GFP fusion protein. The different construct pairs were then introduced into the leaves of *N. benthamiana* via *Agrobacterium*‐mediated transient transformation. After 48 h of infiltration, the Renilla (internal control, REN) and firefly luciferase (Luc) signals were detected with a Dual‐Luc assay kit (Promega, Madison, WI, USA). The primers used for the Dual‐Luc assays were shown in Table S5 (Supporting Information).

### Electrophoretic Mobility Shift Assay

Two wild‐type DNA probes (aAAAAGtgataagaaaaaacatccaagccct and agccagAAAAGaaaatacaataattctcatt) containing a potential SlDOF9 binding motif (AAAAG) were synthesized. The corresponding mutation probes, whose core motifs were mutated, were shown in Table S5 (Supporting Information). The *SlDOF9* and *MBP* coding sequences were cloned into pET28a (+) with a C‐terminal His‐tag. Next, the vectors were transformed into the *Escherichia coli* strain BL21. The recombinant proteins (SlDOF9‐His and MBP‐His) were induced, purified and subjected to SDS‒PAGE (Sodium Dodecyl Sulfate – Polyacrylamide Gel Electrophoresis) electrophoretic analysis. Biotin‐labeled and unlabeled probes were incubated with the purified recombinant protein and anti‐His antibody at room temperature for 30 min. Signals were visualized with the reagents included in the kit and a ChemiDoc XRS (Bio‐Rad Laboratories, Hercules, CA, USA). The SlRR26‐His protein and SlPP2C probe were used as positive controls, as reported in the previous research.^[^ ^29^ ^]^ A negative control was generated with MBP‐His protein and SlSWEET17‐labeled probes.

### ChIP Assay

Leaves of 21‐day‐old SlDOF9‐OE and CM tomato plants were harvested, soaked in 1% formaldehyde, neutralized with 0.125 M glycine and then ground into powder. The chromatin‐bound proteins were isolated, sonicated to break the DNA into 300–500 bp fragments and then incubated with or without anti‐GFP antibody (Abmart, Shanghai, China) for 4 h at 4 °C. Buffer (0.1 M NaHCO_3_ and 0.5% SDS, pH 8.0) was used to elute the immunoprecipitated chromatin. The DNA in the immunoprecipitated chromatin was extracted using a DNA extraction kit (GeneBette, Beijing, China). For the qPCR experiment, the percent input method was used in accordance with a previous study.^[^ ^34^ ^]^ At least three biological replicates were included. The primers used in these experiments were listed in Table S5 (Supporting Information).

### RNA‐Seq Library Construction, Sequencing and Bioinformatics Analysis

Total RNA was isolated from the roots and galls of four wild‐type tomato leaves with or without RKN inoculation using a HiPure Plant RNA Mini Kit (Magen, China). RNA integrity was assessed using the RNA Nano 6000 Assay Kit and the Bioanalyzer 2100 system (Agilent Technologies, Santa Clara, CA, USA). Sequencing libraries, which were generated using the NEB Next Ultra RNA Library Prep Kit for Illumina (New England Biolabs, Ipswich, MA, USA), were sequenced on an Illumina NoveSeq 6000 platform (Illumina, Inc., San Diego, CA, USA) by ORI‐gene Company (Beijing, China). Paired‐end clean reads were aligned to the reference genome (https://solgenomics.net/organism/Solanum_lycopersicum/genome) using HISAT2 (http://ccb.jhu.edu/software/hisat2/index.shtmL, Johns Hopkins University School of Medicine, Baltimore, Maryland, USA). The Fragments Per Kilobase of exon model per Million mapped fragments (FPKM) value was used as an index to measure the expression levels of transcripts or genes. The raw RNA‐seq data have been deposited in the National Genomics Data Center (https://ngdc.cncb.ac.cn/, China, accession no. CRA010849).

The identification of DEGs was performed using DESeq2 (Bioconductor),^[^ ^35^ ^]^ and the list of DEGs was provided in Table S1 (Supporting Information). The criteria for screening the significantly differentially expressed genes were *P* value < 0.01 and ¦log_2_(fold change)¦ > 1.

### High‐Performance Liquid Chromatography (HPLC) Analysis of Sucrose, Glucose and Fructose in Galls

Tomato seedlings with four true leaves were inoculated with RKNs, and galls were harvested at 14 DPI. The sugars in the roots (200‐500 mg fresh weight) were extracted and measured according to previous methods, with some modifications.^[^ ^15^ ^]^ Briefly, the extraction supernatants were evaporated to dryness at 42 °C in a drying oven, dissolved in 0.5–1 mL of deionized water and then filtered. An Agilent 1200 HPLC system (Agilent Technologies, USA) was used to analyze the carbohydrate content. Fifty microliters of 10 mg mL^−1^ sucrose, glucose and fructose standards were gradient diluted to concentrations of 1, 0.5, 0.25, 0.1, and 0.05 mg mL^−1^, respectively, and used as the external standards. Sucrose, glucose, and fructose were identified by comparison with the retention times of known standards and quantified with a refractive index detector (Agilent Technologies, USA). Total sugar contents were calculated as the sum of the sucrose, glucose, and fructose contents.

### Statistical Analysis

The statistical significance of differences between samples was analyzed by Student's *t* test (*P* < 0.05, *P* < 0.01 or *P *< 0.001), and statistical comparisons among the mean values were performed using one‐way analysis of variance (ANOVA), followed by Tukey's multiple range test at the *P* < 0.05 level with GraphPad Prism 8.0 software (GraphPad Software, San Diego, California, USA). The statistical data were provided in Supporting Data Set 1.

### Accession Numbers

Sequence data from this article can be found in the Solanaceae Genomics Network databases under accession numbers: Solyc04g064630 (SlSWEET1c), Solyc06g060590 (SlSWEET1e), Solyc03g097600 (SlSWEET10b), Solyc06g072630 (SlSWEET12d), Solyc01g099870 (SlSWEET17) and Solyc02g090220 (SlDOF9).

## Conflict of Interest

The authors declare no conflict of interest.

## Author Contributions

X.W., Z.W., and X.T. contributed equally to this work. X.W. performed investigation (equal), methodology (equal), visualization (lead), wrote the original draft (lead), wrote reviewed and edited (equal). Z.W. performed investigation (equal), methodology (equal). X.T. performed investigation (equal), methodology (equal). J.Q. performed methodology (supporting). X.Z. performed investigation (supporting), and methodology (supporting). L.G. performed methodology (supporting). H.B. performed methodology (supporting). L.S. performed methodology (supporting), and provided resources (supporting). H.H. performed methodology (supporting), wrote, reviewed and edited (supporting). R.Y. performed methodology (supporting). J.W. provided resources (supporting). S.W. performed funding acquisition (equal), project administration (equal), and supervision (equal). S.C. performed methodology (supporting). Z.Y. performed methodology (supporting). W.Z. performed conceptualization (lead), Funding acquisition (equal), Project administration (equal), Supervision (equal), wrote the original draft (supporting), wrote‐reviewed and edited (equal).

## Supporting information



Supporting Information

Supporting Information

Supporting Information

## Data Availability

The data that support the findings of this study are available in the supplementary material of this article.

## References

[1] Y. Chen , A. J. Miller , B. Qiu , Y. Huang , K. Zhang , G. Fan , X. Liu , Plant Biotechnol. J. 2024, 22, 2844. 38879813 10.1111/pbi.14408 PMC11536462

[2] K. Yamada , Y. Saijo , H. Nakagami , Y. Takano , Science 2016, 354, 1427. 27884939 10.1126/science.aah5692

[3] W. B. Rutter , J. Franco , C. Gleason , Annu. Rev. Phytopathol. 2022, 60, 43. 35316614 10.1146/annurev-phyto-021621-120943

[4] S. Siddique , F. M. W. Grundler , Curr. Opin. Microbiol. 2018, 46, 102. 30326406 10.1016/j.mib.2018.09.004

[5] M. Naseem , M. Kunz , T. Dandekar , Trends Plant Sci. 2017, 22, 740. 28779901 10.1016/j.tplants.2017.07.001

[6] L. Q. Chen , New Phytologist 2013, 201, 1150. 24649486 10.1111/nph.12445

[7] a) L.‐Q. Chen , B.‐H. Hou , S. Lalonde , H. Takanaga , M. L. Hartung , X.‐Q. Qu , W.‐J. Guo , J.‐G. Kim , W. Underwood , B. Chaudhuri , D. Chermak , G. Antony , F. F. White , S. C. Somerville , M. B. Mudgett , W. B. Frommer , Nature 2010, 468, 527; 21107422 10.1038/nature09606 PMC3000469

[8] Y. H. Xuan , Y. B. Hu , L.‐Q. Chen , D. Sosso , D. C. Ducat , B.‐H. Hou , W. B. Frommer , Proc. Natl. Acad. Sci. USA 2013, 110, E3685. 24027245 10.1073/pnas.1311244110 PMC3785766

[9] a) M. Cohn , R. S. Bart , M. Shybut , D. Dahlbeck , M. Gomez , R. Morbitzer , B.‐H. Hou , W. B. Frommer , T. Lahaye , B. J. Staskawicz , Mol. Plant‐Microbe Interact. 2014, 27, 1186; 25083909 10.1094/MPMI-06-14-0161-R

[10] a) M. Andargie , J. Li , J. Plant Biochem. Biotechnol. 2019, 28, 509;

[11] a) Y. Zhou , D. Zhao , Y. Duan , L. Chen , H. Fan , Y. Wang , X. Liu , L.‐Q. Chen , Y. Xuan , X. Zhu , Front. Plant Sci. 2023, 14, 1010348; 36824200 10.3389/fpls.2023.1010348 PMC9941640

[12] W. Sun , Z. Gao , J. Wang , Y. Huang , Y. Chen , J. Li , M. Lv , J. Wang , M. Luo , K. Zuo , New Phytol. 2019, 222, 864. 30506685 10.1111/nph.15620

[13] X. Li , W. Guo , J. Li , P. Yue , H. Bu , J. Jiang , W. Liu , Y. Xu , H. Yuan , T. Li , A. Wang , Plant Physiol. 2020, 182, 2035. 32047049 10.1104/pp.20.00002 PMC7140945

[14] a) Y. Wu , S.‐K. Lee , Y. Yoo , J. Wei , S.‐Y. Kwon , S.‐W. Lee , J.‐S. Jeon , G. An , Mol. Plant 2018, 11, 833; 29656028 10.1016/j.molp.2018.04.002

[15] Y. Wang , H. Zhao , L. Xu , H. Zhang , H. Xing , Y. Fu , L. Zhu , New Phytol. 2022, 237, 1667. 36444526 10.1111/nph.18635

[16] J. Singh , S. Das , K. J. Gupta , A. Ranjan , C. H. Foyer , J. K. Thakur , Plant Biotechnol. J. 2023, 21, 1528. 36529911 10.1111/pbi.13982 PMC10363763

[17] T. Aprees , Curr. Biol. 1994, 4, 557.7922381

[18] G. Hu , K. Wang , B. Huang , I. Mila , P. Frasse , E. Maza , A. Djari , M. Hernould , M. Zouine , Z. Li , M. Bouzayen , Nat. Plants 2022, 8, 419. 35422080 10.1038/s41477-022-01121-1

[19] J. Ji , L. Yang , Z. Fang , Y. Zhang , M. Zhuang , H. Lv , Y. Wang , Biomolecules 2022, 12, 205. 35204707 10.3390/biom12020205 PMC8961523

[20] Y.‐F. Guan , X.‐Y. Huang , J. Zhu , J.‐F. Gao , H.‐X. Zhang , Z.‐N. Yang , Plant Physiol. 2008, 147, 852. 18434608 10.1104/pp.108.118026 PMC2409014

[21] M. L. Engel , R. Holmes‐Davis , S. McCormick , Plant Physiol. 2005, 138, 2124. 16055690 10.1104/pp.104.054213 PMC1183400

[22] Y. X. Ge , G. C. Angenent , P. E. Wittich , J. Peters , J. Franken , M. Busscher , L. M. Zhang , E. Dahlhaus , M. M. Kater , G. J. Wullems , T. Creemers‐Molenaar , Plant J. 2000, 24, 725. 11135107 10.1046/j.1365-313x.2000.00926.x

[23] a) M. Valifard , R. Le Hir , J. Müller , D. Scheuring , H. E. Neuhaus , B. Pommerrenig , Plant Physiol. 2021, 187, 2716; 34597404 10.1093/plphys/kiab436 PMC8644896

[24] X. Zhang , C. Feng , M. Wang , T. Li , X. Liu , J. Jiang , Hortic. Res. 2021, 8, 123. 34333539 10.1038/s41438-021-00624-w PMC8325691

[25] a) A. Anjali , U. Fatima , M. S. Manu , S. Ramasamy , M. Senthil‐Kumar , Plant Physiol. Biochem. 2020, 156, 1; 32891967 10.1016/j.plaphy.2020.08.043

[26] Y. Xu , Y. Tao , L. S. Cheung , C. Fan , L.‐Q. Chen , S. Xu , K. Perry , W. B. Frommer , L. Feng , Nature 2014, 515, 448. 25186729 10.1038/nature13670 PMC4300204

[27] G. Antony , J. Zhou , S. Huang , T. Li , B. Liu , F. White , B. Yang , Plant Cell 2010, 22, 3864. 21098734 10.1105/tpc.110.078964 PMC3015117

[28] D. Zhao , Y. You , H. Fan , X. Zhu , Y. Wang , Y. Duan , Y. Xuan , L. Chen , Int. J. Mol. Sci. 2018, 19, 302 29351253 10.3390/ijms19010302 PMC5796247

[29] W. Zhao , J. Liang , H. Huang , J. Yang , J. Feng , L. Sun , R. Yang , M. Zhao , J. Wang , S. Wang , New Phytol. 2023, 238, 1651. 36829301 10.1111/nph.18837

[30] X. Li , W. Zhao , X. Zhou , J. Feng , Y. Gao , X. Yao , Y. Liu , J. Liu , R. Yang , F. Zhao , S. Wang , Hortic. Environ. Biotechnol. 2017, 58, 620.

[31] a) C. An , L. Deng , H. Zhai , Y. You , F. Wu , Q. Zhai , A. Goossens , C. Li , Mol. Plant 2022, 15, 1329; 35780296 10.1016/j.molp.2022.06.014

[32] H.‐L. Xing , L. Dong , Z.‐P. Wang , H.‐Y. Zhang , C.‐Y. Han , B. Liu , X.‐C. Wang , Q.‐J. Chen , BMC Plant Biol. 2014, 14, 327. 25432517 10.1186/s12870-014-0327-y PMC4262988

[33] T. Ho‐Plágaro , R. Huertas , M. I. Tamayo‐Navarrete , J. A. Ocampo , J. M. García‐Garrido , Plant Methods 2018, 14, 34. 29760765 10.1186/s13007-018-0304-9 PMC5941616

[34] N. Yamaguchi , C. M. Winter , M.‐F. Wu , C. S. Kwon , D. A. William , D. Wagner , Arabidopsis Book 2014, 12, 0170. 10.1199/tab.0170 PMC3952383 24653666

[35] M. I. Love , W. Huber , S. Anders , Genome Biol. 2014, 15, 550. 25516281 10.1186/s13059-014-0550-8 PMC4302049

