# Supplementary material for: The SlDOF9‐SlSWEET17 Module: a Switch for Controlling Sugar Distribution Between Nematode Induced Galls and Roots in Tomato
Source: Adv Sci (Weinh). 2025 May 11;12(28):2501771. doi: 10.1002/advs.202501771 (PMC12302606; doi:10.1002/advs.202501771)
Supplement: Supplementary file 1 — Supporting Information [file ADVS-12-2501771-s001.pdf]

ADVANCED SCIENCE
Open Access

Supporting Information

for *Adv. Sci.*, DOI 10.1002/advs.202501771

The SlDOF9-SlSWEET17 Module: a Switch for Controlling Sugar Distribution Between Nematode Induced Galls and Roots in Tomato

*Xiaoyun Wang, Zhimei Wang, Xinyue Tang, Jiamei Qin, Xiaoxuan Zhou, Lixia Gu, Huihui Bian, Lulu Sun, Huang Huang, Rui Yang, Jianli Wang, Shaohui Wang, Shuangchen Chen, Zhongren Yang and Wenchao Zhao**

Supporting Information

The SlDOF9-SlSWEET17 module: A switch for controlling sugar distribution between nematode induced galls and roots in tomato

*Xiaoyun Wang, Zhimei Wang, Xinyue Tang, Jiamei Qin, Xiaoxuan Zhou, Lixia Gu, Huihui Bian, Lulu Sun, Huang Huang, Rui Yang, Jianli Wang, Shaohui Wang, Shuangchen Chen, Zhongren Yang and Wenchao Zhao**

Supporting Information List：

File name	Contents
**Supplemental Figures 1-8**	Figure S1. Subcellular localization of SlSWEET17 in galls.
	Figure S2. Highly expressed *SWEET* genes in galls.
	Figure S3. SlSWEETs localized to the plasma membrane.
	Figure S4. Co-subcellular localization of SlSWEETs with or without SlSWEET17.
	Figure S5. Negative control of BiFC.
	Figure S6. The expression of the four SlSWEET genes in different lines.
	Figure S7. Subcellular localization and the relative expression level of SlDOF9.
	Figure S8. The expression of SlSWEET17 and SlDOF9 in $sweet17^{cr}dof9^{cr}$ plant.
**Supplemental Tables 1-5**	Supplemental Table 1. Differentially expressed genes between control and galls from RNA-seq data.
	Supplemental Table 2. Promoter analysis of SlSWEET17 by PlantPAN 4.0.
	Supplemental Table 3. Cis-acting element family analysis of SlSWEET17 promoter prediction results.
	Supplemental Table 4. The most predicted binding sites of DOF transcription factors in SlSWEET17 promoter.
	Supplemental Table 5. Primers used in this study.
**Supplemental Dataset**	Supplemental Dataset 1. Statistic analysis

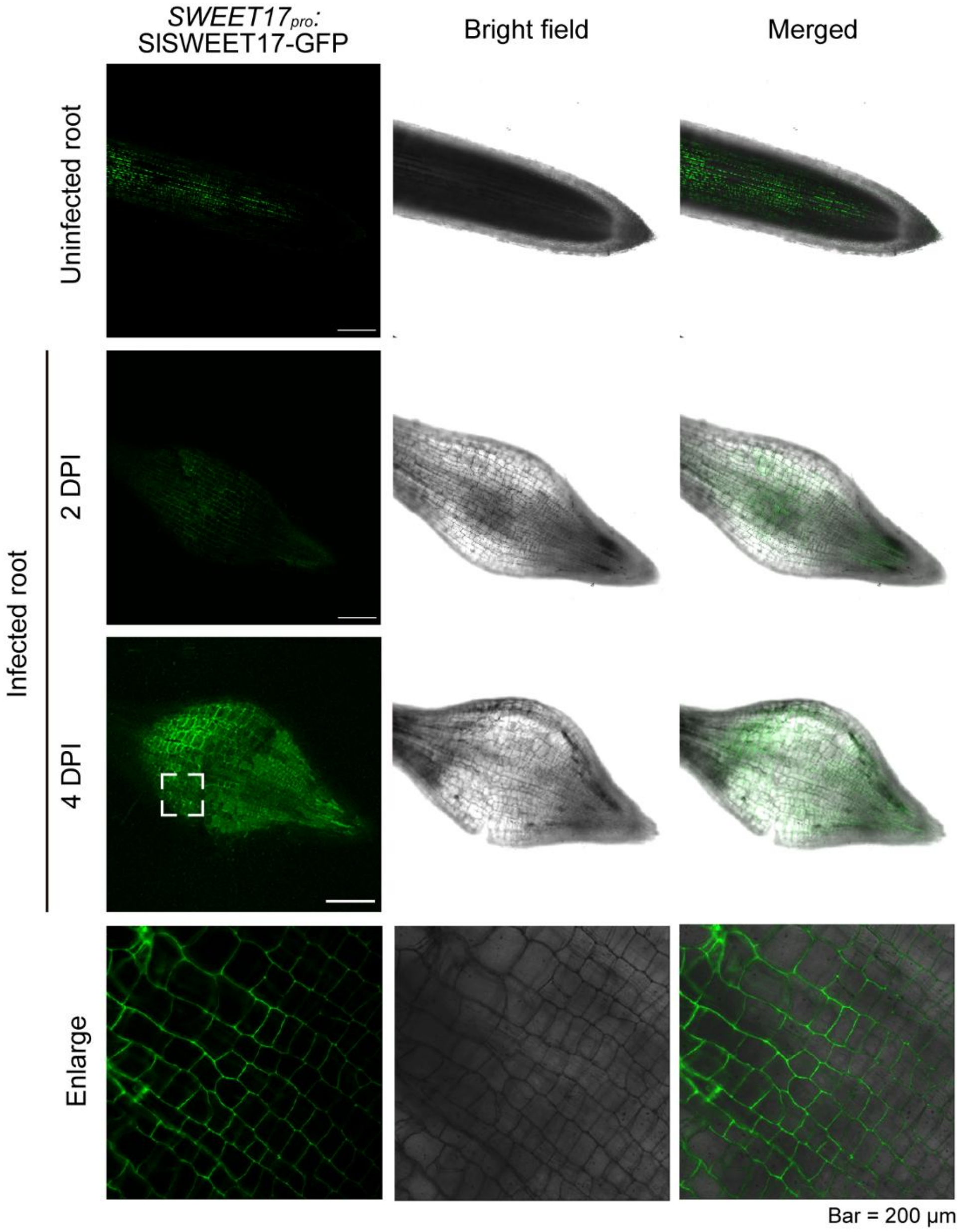

Figure S1. Subcellular localization of SlSWEET17 in galls.

Subcellular localization of SlSWEET17 protein expression driven by the SlSWEET17 promoter (*SWEET17$_{pro}$:SlSWEET17-GFP*) in hairy roots and galls. Hairy roots with galls were observed at 2 DPI and 4 DPI. Yellow bar = 200 μm, white bar = 40 μm. (SWEET, sugars will eventually be exported transporters; GFP, green fluorescence protein; DPI, day post inoculation).

Gene Name	Gene ID	Relative Expression in Gall (FPKM)
SlSWEET1c	**Solyc04g064630.2**	**30.885**
SlSWEET1e	**Solyc06g060590.2**	**13.7489**
SlSWEET10b	**Solyc03g097600.2**	**9.54696**
SlSWEET12d	**Solyc06g072630.2**	**5.97905**
SlSWEET17	Solyc01g099870.1	4.6907
SlSWEET2b	Solyc07g062120.2	3.72002
SlSWEET10a	Solyc03g097580.2	3.59279
SlSWEET11b	Solyc03g097570.2	2.66116
SlSWEET11c	Solyc06g072620.2	2.56284
SlSWEET12c	Solyc05g024260.2	1.80822
SlSWEET10c	Solyc03g097610.2	1.44811
SlSWEET12a	Solyc03g097590.2	1.43011
SlSWEET7a	Solyc08g082770.2	1.15223
SlSWEET3	Solyc03g007360.2	1.12371
SlSWEET1d	Solyc04g064640.2	0.980959
SlSWEET12b	Solyc03g097620.1	0.601963
SlSWEET14	Solyc03g097560.2	0.59067
SlSWEET1f	Solyc06g060580.1	0.387099
SlSWEET16	Solyc01g099880.2	0.30191

Figure S2. Highly expressed *SWEET* genes in galls.

The expression of *SWEET* genes in gall were tested by RNA-Seq. (SWEET, sugars will eventually be exported transporters; RNA-Seq, RNA sequencing; FPKM, fragments per kilobase of transcript per million fragments mapped).

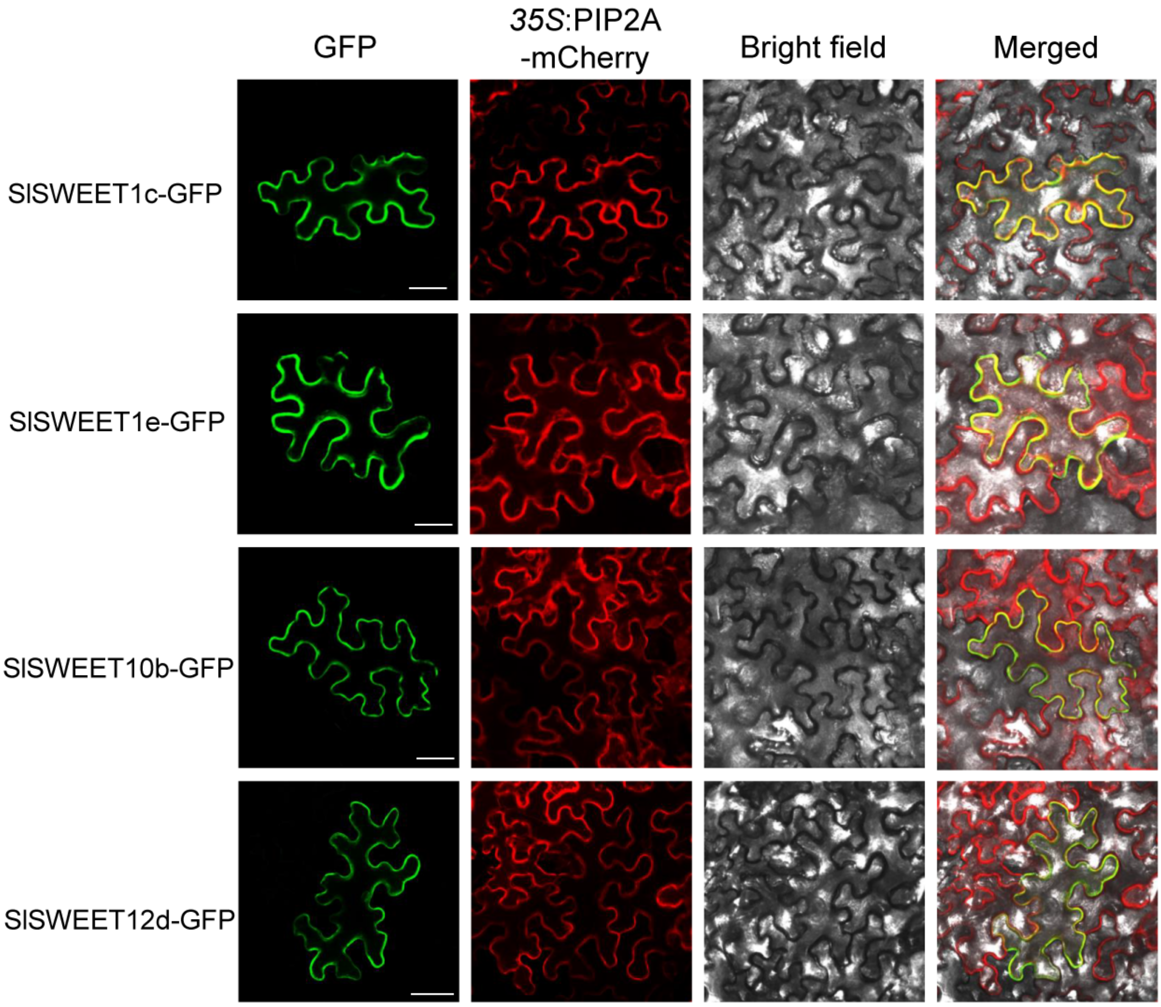

Figure S3. SlSWEETs localized to the plasma membrane.

Subcellular localization of SlSWEETs driven by *Super* promoter in leaf epidermal cells of *N. benthamiana*. *35S:*PIP2A-mCherry was used as plasma membrane marker. Bar = 40 µm. (SWEET, sugars will eventually be exported transporters; PIP2A, plasma membrane intrinsic protein 2A; GFP, green fluorescence protein).

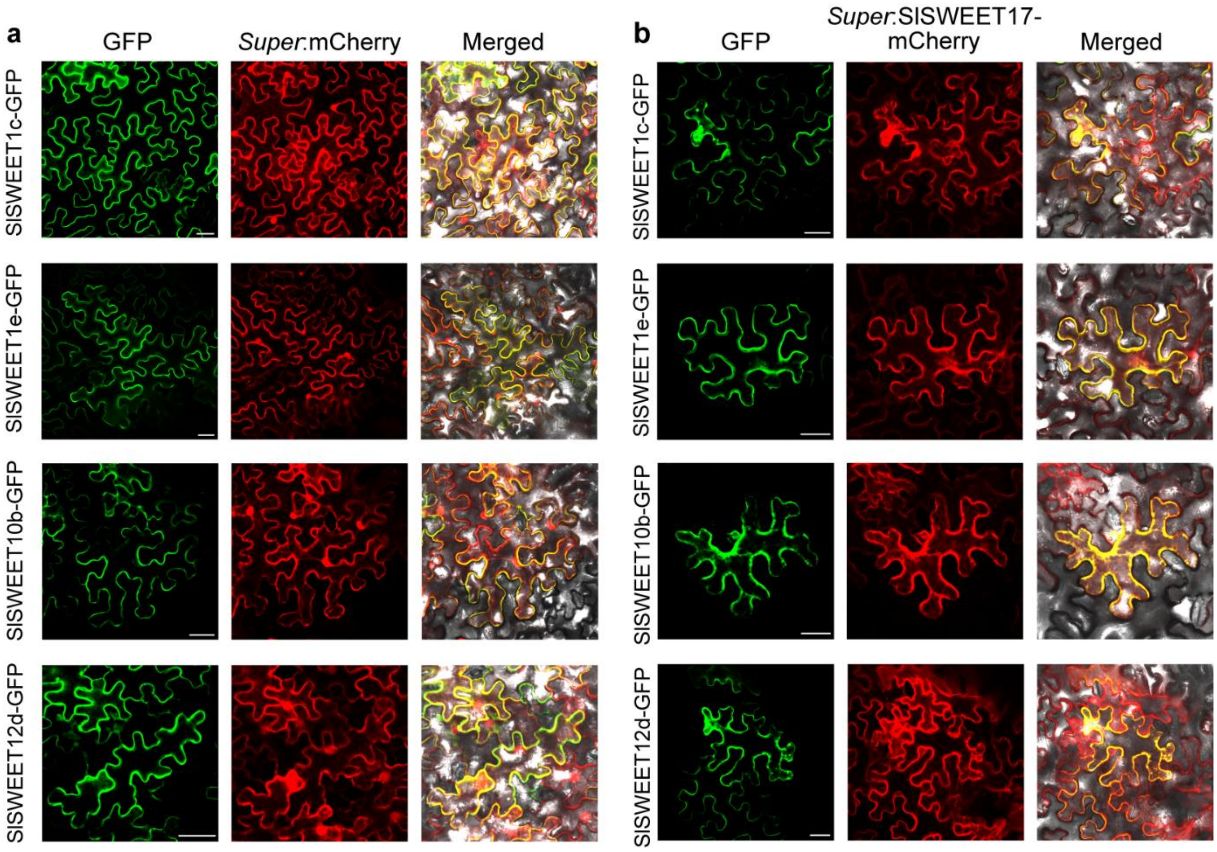

Figure S4. Co-subcellular localization of SlSWEETs with or without SlSWEET17. SlSWEETs proteins were driven by *Super* promoter in leaf epidermal cells of *N. benthamiana*. **a,** Co-subcellular localization of SlSWEETs with *Super:*mCherry as negative control. **b,** Co-subcellular localization of SlSWEETs with *Super:*SlSWEET17-mCherry. Bar = 40 µm. (SWEET, sugars will eventually be exported transporters; GFP, green fluorescence protein).

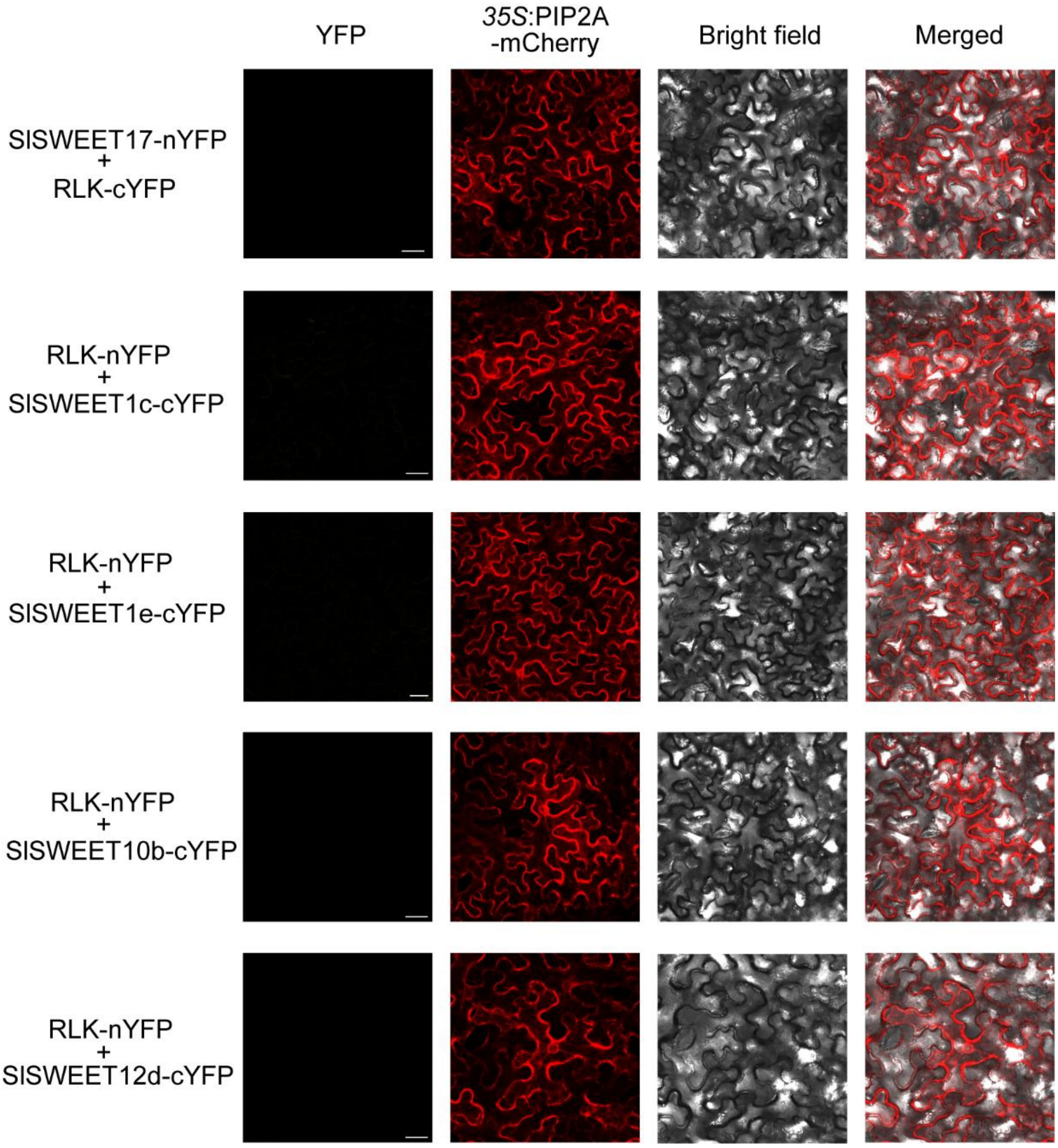

Figure S5. Negative control of BiFC.

Tomato membrane protein-localized receptor-like kinase (RLK) with nYFP or cYFP were co-transformed with nYFP-SlSWEET17 or cYFP-SlSWEETs to serve as negative controls of BiFC. *35S:*PIP2A-mCherry was used as plasma membrane marker. Bars = 40 μm. (SWEET, sugars will eventually be exported transporters; YFP, yellow fluorescence protein).

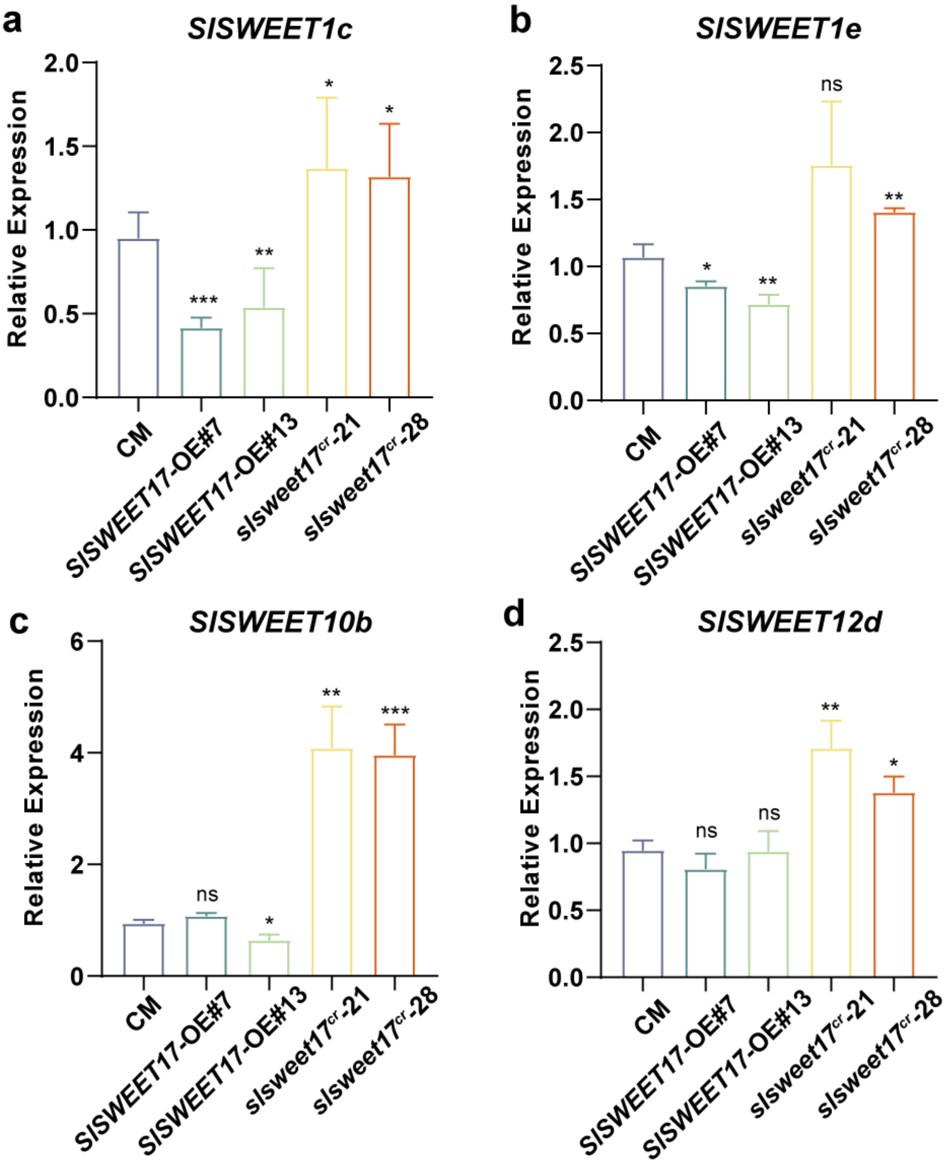

Figure S6. The expression of the four *SlSWEET* genes in different lines.

The relative expression of *SlSWEET1c* (**a**), *SlSWEET1e* (**b**), *SlSWEET10b* (**c**) and *SlSWEET12d* (**d**) in *SlSWEET17*-OE, *slsweet17*^{cr} and CM plants. Values are the mean ±SD of at least three biological replicates. Asterisk represents significant differences (*, $P < 0.05$; **, $P < 0.01$; ***, $P < 0.001$) by unpaired T test. (SWEET, sugars will eventually be exported transporters; CM, tomato cultivar Castlemart).

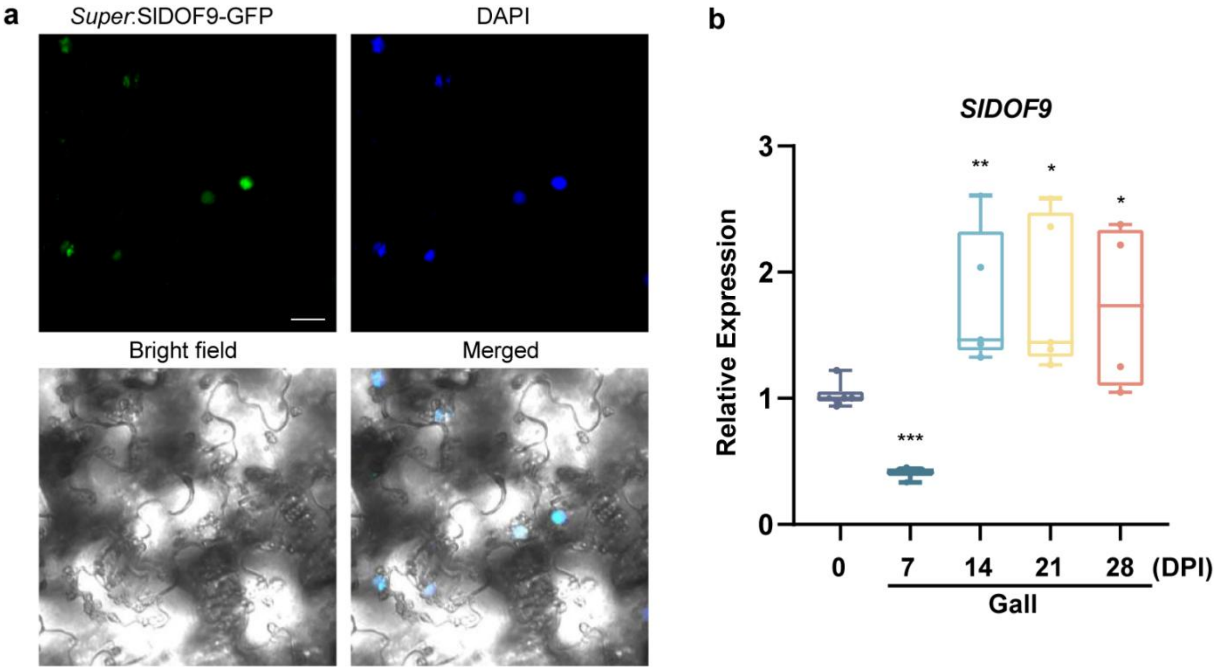

Figure S7. Subcellular localization and the relative expression level of SlDOF9.

a, Subcellular localization of SlDOF9 driven by *Super* promoter in leaf epidermal cells of *N. benthamiana*. DAPI staining was used as nuclear marker. Bars = 40 µm. **b,** The relative expression of *SlDOF9* of multiple time points (including 0, 7,14,21,28 DPI) during RKN infection. Values are the mean ±SE of at least four biological replicates. Asterisk represents significant differences (*, $P < 0.05$; **, $P < 0.01$; ***, $P < 0.001$) by unpaired T test. (DOF, DNA binding with one finger; GFP, green fluorescence protein; DPI, day post inoculation).

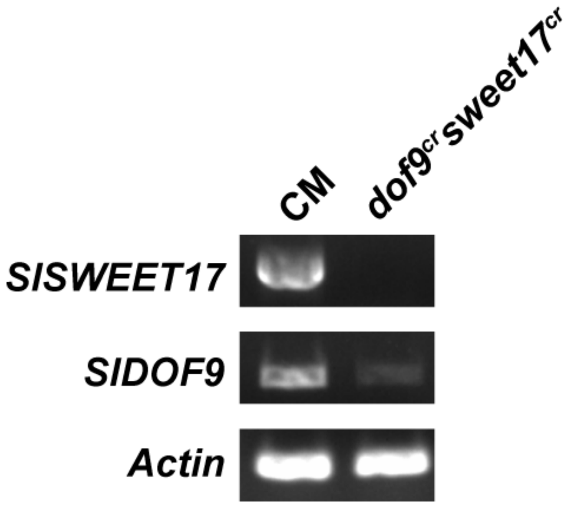

Figure S8. The expression of SlSWEET17 and SlDOF9 in *sweet17^{cr}dof9^{cr}* plant.

The expression of SlSWEET17 and SlDOF9 mRNA verified with end-point RT-PCR (reverse transcription polymerase chain reaction). SlActin (Solyc11g005330) was used as a control of cDNA quality. (SWEET, sugars will eventually be exported transporters; DOF, DNA binding with one finger; CM, tomato cultivar Castlemart).
